# PGPR-Treated Spent Mushroom Substrate Enhances Lignocellulose Degradation, Enzyme Activities, and Microbial Restructuring to Sustain Blueberry Rhizosphere Fertility

**DOI:** 10.3390/microorganisms14081827

**Published:** 2026-08-18

**Authors:** Mengjiao Wang, Ningqiang Li, Yinku Liang, Zhimin Xu, Haicui Wu

**Affiliations:** 1 School of Biological Science and Engineering, Shaanxi University of Technology, Hanzhong 723001, China; m1255413485@163.com (N.L.); liangyinku26@163.com (Y.L.); 2 State Key Laboratory of Qinba Resource and Ecological Environment Jointly Established by the Ministry and Ministry of Education (Cultivation), Shaanxi University of Technology, Hanzhong 723001, China; 3 Shaanxi Key Laboratory of Bioresources, Shaanxi University of Technology, Hanzhong 723000, China; 4 School of Nutrition and Food Sciences, Louisiana State University, Baton Rouge, LA 70802, USA; 5 Department of Food Science and Nutrition, The Hong Kong Polytechnic University, Hung Hom, Kowloon, Hong Kong, China; haicui123.wu@polyu.edu.hk

**Keywords:** spent mushroom substrate, plant growth-promoting rhizobacteria, blueberry, rhizosphere microbiome, soil enzymes, lignocellulose degradation

## Abstract

Spent mushroom substrate (SMS) is a major agricultural byproduct whose complex lignocellulosic matrix hinders direct reuse and poses environmental risks when stockpiled. This study evaluated whether pretreatment with plant growth-promoting rhizobacteria (PGPR) could enhance SMS as a soil amendment for blueberry cultivation. Two PGPR-treated SMS formulations, along with raw SMS and a blank control, were applied to blueberry seedlings in a 10-month greenhouse experiment. Plant height, rhizosphere soil nutrients, enzyme activities, lignocellulose fractions, and the microbial communities were monitored over three growth phases and four sampling points. PGPR-treated SMS significantly increased blueberry height gain during the fast-growing phase (June–September) and sustained elevated levels of organic carbon, nitrogen, phosphorus, and potassium throughout the experiment. Activities of cellulase, xylanase, laccase, peroxidase, protease, and lipase were markedly enhanced, accompanied by reduced lignin and cellulose contents and persistently high glucose availability. The amendments reshaped bacterial and fungal communities, enriching *Bacillota*, *Acidobacteriota*, *Acidibacter*, and *Hyphomicrobium*, and increasing alpha diversity, with clear structural separation from controls in principal coordinate analysis. Correlation and principal component analyses linked improved plant growth to nutrient availability, enzyme stimulation, and specific microbial taxa. These findings indicate that PGPR-treated SMS acts as a multifunctional amendment that promotes lignocellulose degradation, sustains soil fertility, and restructures the rhizosphere microbiome, offering a sustainable recycling strategy for horticultural production.

## 1. Introduction

Edible mushrooms are widely regarded as “next-generation food” owing to their high nutritional value and biological functionality [1]. Spent mushroom substrate (SMS), the residual biomass remaining after the harvest of edible fungal fruitbodies, is a significant byproduct of mushroom cultivation [2]. The production of 1 kg of fresh mushrooms yields approximately 5 kg of wet SMS [3]. Global production projections estimate 2024 yields at 15.0–15.5 million metric tons, and the associated SMS production reaches 75.0–77.5 million metric tons annually, necessitating the urgent development of valorization strategies to address environmental concerns from this lignocellulosic byproduct [4,5]. The efficient recycling and valorization of SMS are critical for advancing the sustainable development of the mushroom industry according to the principles of a circular economy [6]. Finding effective ways to reuse SMS is therefore an urgent task.

To meet this need, researchers have explored several ways to reuse SMS. SMS is composed of residual mushroom mycelium and the growth substrate, which typically includes materials such as cottonseed hulls, wood chips, wheat bran, corn cobs, straw, and other cellulose-rich components [7]. This versatile byproduct has been used for many purposes. For example, it serves as animal feed to improve gut health and livestock growth [8]. It has also been tested as a raw material for making biofuels like biogas and ethanol [9]. Among these various options, using SMS as a soil conditioner stands out as a particularly promising approach. SMS is added to soil because it has good air flow, holds water well, retains nutrients, and has a loose structure [10]. These properties make it an attractive choice for improving soil health. However, when SMS is used directly as a soil conditioner, plants cannot easily break it down or use its nutrients. This is because SMS has a tough structure made of lignin and cellulose that resists decomposition. Therefore, finding ways to speed up SMS breakdown and release its nutrients is important for making better use of it in farming.

Plant growth-promoting rhizobacteria (PGPR) are a group of plant-associated microorganisms known for their ability to enhance plant growth, improve disease resistance, and boost soil quality [11]. PGPR strains have been widely utilized as microbial starters for fermenting industrial and agricultural waste [12]. For instance, PGPR have been used to treat crop straw and other cellulose-rich agricultural residues. These treatments help break down tough plant fibers and turn them into nutrients that plants can use [13,14,15]. In recent years, researchers have also started to apply PGPR to treat spent mushroom substrate [16]. Several studies have demonstrated the effectiveness of this approach. For example, Wang et al. used three PGPR strains to treat SMS and found that after 54 days of decomposition, the treated SMS showed significantly higher total nitrogen, hydrolysable nitrogen, and available phosphorus compared to untreated controls. When this material was mixed with soil for blueberry cultivation, it significantly improved plant growth, soil nutrient content, and rhizosphere microbial diversity [16]. In another study, Li et al. combined spent mushroom substrate from *Hypsizygus marmoreus* with the PGPR strain *Rhizobium pusense* through solid-state fermentation [17]. When 40% of this fermented material was added to soil, it increased total nitrogen, ammonium nitrogen, and organic matter content, decreased electrical conductivity, raised pH, and significantly improved soil enzyme activities (catalase, dehydrogenase, and urease) [17]. Similarly, Fan et al. studied the combined application of PGPR and SMS in ginseng cultivation and found that the combination treatment significantly increased soil enzyme activities (urease by 7.29%, sucrase by 29.76%, acid phosphatase by 13.24%, and amylase by 38.25%), enriched beneficial bacteria such as *Allorhizobium-Neorhizobium-Pararhizobium-Rhizobium*, and reduced harmful bacteria like *Klebsiella* and the pathogenic fungus *Fusarium* [18]. Beyond these pot experiments, PGPR have also been tested in composting systems. Zhang et al. inoculated SMS compost with *Bacillus paralicheniformis* and *Streptomyces thermoviolaceus*. The inoculation extended the high-temperature phase of composting by 2–3 days and increased the degradation rates of cellulose, hemicellulose, and lignin by 18–30%, 36–50%, and 40–41%, respectively, compared to the uninoculated control [19]. Inoculation also rapidly altered the microbial community structure in the early stage of composting and strengthened microbial interactions [19]. These studies collectively show that PGPR can accelerate SMS decomposition and release nutrients that plants can use.

Despite these promising results, several important questions remain unanswered. Most of these studies have focused on short-term effects or single indicators such as nutrient content or plant growth. Few studies have examined how PGPR-treated SMS affects soil properties over time. Little is known about its effects on soil enzyme activities, lignocellulose breakdown, and the root-associated microbial community during different growth stages of the crop. In particular, information on blueberry cultivation is still very limited.

Blueberry (*Vaccinium corymbosum* L.) is a globally important fruit crop with high economic value and growing consumer demand. However, blueberry cultivation is particularly sensitive to soil conditions. Unlike most crop plants, blueberries have a shallow, fibrous root system that lacks root hairs, making them highly dependent on soil physicochemical properties and rhizosphere microbial communities for nutrient and water uptake. They require acidic soils (pH 4.5–5.5) with high organic matter content, good drainage, and adequate moisture for optimal growth and development. The availability of soil nutrients and the activity of rhizosphere microorganisms are critical factors influencing blueberry productivity. These characteristics make blueberry an ideal model plant for evaluating the effectiveness of organic soil amendments. Specifically, because blueberries are particularly responsive to changes in soil organic matter and microbial activity, they serve as a sensitive indicator for assessing the functional benefits of PGPR-treated SMS as a soil amendment.

In our previous studies, we demonstrated that the selected PGPR strains exhibit significant growth-promoting effects on blueberry seedlings and can substantially improve rhizosphere soil quality [16,20]. Given the high sensitivity of blueberry to soil conditions and its economic importance, we selected it as the sole test plant to conduct an in-depth, temporally resolved investigation into the mechanisms by which PGPR-treated SMS influences rhizosphere processes. Building on these findings, the present study was designed to further investigate the effects of PGPR-treated SMS on the temporal dynamics of soil fertility, enzyme activities, lignocellulose degradation, and microbial community restructuring in the blueberry rhizosphere. To fill these knowledge gaps, the overarching aim of this study was to comprehensively evaluate the efficacy of PGPR-treated SMS as a multifunctional soil amendment for sustainable blueberry cultivation. We focused on a single crop species to allow for detailed mechanistic analysis across multiple time points and to avoid confounding factors that would arise from interspecific variation in root physiology and rhizosphere interactions. This single-species approach enables us to establish a robust mechanistic framework for how PGPR-treated SMS improves rhizosphere fertility, which can later be extended to other horticultural crops in future comparative studies.

To address this aim, we treated spent mushroom substrate with two PGPR strains (T1 and T2) and applied the decomposed material to blueberry seedlings. We monitored plant growth over three growth periods (June–September, October–December, and January–March). We collected rhizosphere soil samples at four time points and analyzed changes in soil nutrients, enzyme activities, lignocellulose components, and microbial community structure. The specific objectives of this study are: (1) to evaluate the effects of PGPR-treated SMS on blueberry height increment across different growth periods; (2) to determine changes in rhizosphere soil nutrient contents and physicochemical properties under different treatments; (3) to assess soil enzyme activities and lignocellulose degradation dynamics; and (4) to characterize the shifts in bacterial and fungal community composition and diversity in the blueberry rhizosphere over time. These findings will provide a comprehensive understanding of the temporal dynamics of soil–plant–microbe interactions under PGPR-amended SMS. They will also offer a practical basis for recycling spent mushroom substrate in blueberry cultivation and support the development of sustainable soil management strategies in horticultural production.

## 2. Materials and Methods

### 2.1. PGPR Strain Preparation and SMS Treatment

SMS was air-dried, ground (200 rpm, 10 min), and sieved through a 20-mesh screen (0.85 mm). The SMS was sterilised by autoclaving at 121 °C for 30 min prior to use. The two PGPR strains (designated T1 and T2) were previously isolated from the rhizosphere soil of healthy blueberry plants (*Vaccinium corymbosum* L.) in our laboratory and taxonomically identified by 16S rRNA gene sequencing as *Pseudomonas* sp. (T1) and *Buttiauxella* sp. (T2), as described in our previous study [20]. The plant growth-promoting properties of both strains, including indole-3-acetic acid (IAA) production, phosphate solubilization, and siderophore secretion, were confirmed in our earlier work [20]. For inoculum preparation, each strain was separately cultured in Luria–Bertani (LB) liquid medium (containing 10 g/L tryptone, 5 g/L yeast extract, and 10 g/L NaCl, pH 7.0) at 28 °C for 72 h with shaking at 180 rpm in 250 mL Erlenmeyer flasks. After incubation, the bacterial cultures reached a cell density of approximately 1 × 10^8^ CFU/mL, as determined by the plate count method. The resulting liquid cultures (containing both bacterial cells and spent culture medium) were directly mixed with sterilised SMS and sterile distilled water at a ratio of 1:3:10 (*v*:*w*:*v*). The initial inoculation density in the mixture was thus approximately 1 × 10^7^ CFU per gram of dry SMS. The mixture was incubated at 28 °C for 3 days, followed by aerobic decomposition at 25 °C for 54 days [16]. Treated SMS was mixed with peat-based nutrient soil (Brighten 2024, a commercial peat-based substrate with pH 5.5–6.0, Pindstrup Group, Ryomgård, Denmark) at a 1:4 ratio (SMS: nutrient soil, *v*/*v*) to form T1 and T2 substrates, respectively. Controls were pure nutrient soil (CKS) and sterilised SMS mixed with the same nutrient soil at the same 1:4 ratio (CKM). Raised beds (6 m^2^, 40 cm depth) were used in a randomised complete block design with three replicates in a greenhouse. One-year-old blueberry plants (*Vaccinium corymbosum* L.) were transplanted on 15 May 2025. Standard agronomic practices were applied uniformly. A schematic overview of the experimental design is provided in Figure S1. Detailed procedures are provided in Text S1.

### 2.2. Sample Collection and Determination of Nutritional Elements in Rhizosphere Soil

Rhizosphere soil was sampled at four time points (15 June, 15 September, 15 December 2025, and 15 March 2026) following the procedure of Wang et al. [20,21]. Samples were air-dried, ground, and sieved (2 mm). All analyses were in triplicate. Organic carbon (OC) was measured by K_2_Cr_2_O_7_ oxidation [22], total nitrogen (TN) by Kjeldahl [23], hydrolysis nitrogen (HN) by alkaline hydrolysis [24], total phosphorus (TPH) by H_2_SO_4_-HClO_4_ digestion molybdenum blue colorimetry [25], available phosphorus (APH) by Olsen method [26], total potassium (TPO) by atomic emission spectrometry [27], available potassium (APO) by flame photometry [28], exchangeable Na^+^, Ca^2+^, Mg^2+^ by BaCl_2_ extraction and flame photometry [29], electrical conductivity (EC) by a portable meter [30], and moisture by oven-drying [31]. All detailed procedures are provided in Text S2.

### 2.3. Determination of Enzyme Activities and Biochemical Components in Rhizosphere Soil

Cellulase and xylanase activities were assayed by the DNS method [31]. Laccase activity was determined by ABTS oxidation [32]. Peroxidase activity was measured by pyrogallol [33]. Protease activity was assayed using casein–Folin–Ciocalteu method [34]. Lipase activity was determined using p-nitrophenyl palmitate (pNPP) [35]. Lignin, hemicellulose, and cellulose contents were determined by the Van Soest detergent system [36]. Glucose was quantified by HPAEC-PAD [37]. Lignin phenolic hydroxyl ratio was measured using CuO oxidation followed by GC analysis of phenolic monomers [38], and the ratio was calculated relative to a standard lignin preparation [39]. All enzyme and biochemical assays were conducted on fresh or processed soil samples with three replicates. All detailed procedures are provided in Text S3.

### 2.4. Soil Microbial Community Analysis

Total genomic DNA was extracted from 0.5 g of rhizosphere soil using the FastDNA Spin Kit for Soil (MP Biomedicals, Irvine, CA, USA) following the manufacturer’s instructions. The V4 region of the bacterial 16S rRNA gene was amplified with primers 515F (5′-GTGYCAGCMGCCGCGGTAA-3′) and 806R (5′-GGACTACNVGGGTWTCTAAT-3′) [40], and fungal ITS2 with ITS3 (5′-GCATCGATGAAGAACGCAGC-3′) and ITS4 (5′-TCCTCCGCTTATTGATATGC-3′) [41]. PCR amplifications were performed in a Bio-Rad S1000 Thermal Cycler (Bio-Rad Laboratories, Hercules, CA, USA) with the following protocol: initial denaturation at 95 °C for 3 min; 30 cycles of denaturation at 95 °C for 30 s, annealing at 55 °C for 30 s, and extension at 72 °C for 45 s; and a final extension at 72 °C for 10 min. Amplicons were purified, quantified, pooled and sequenced on an Illumina HiSeq 2500 platform (Illumina, San Diego, CA, USA) [42]. Raw reads were processed with the DADA2 pipeline within QIIME2 (version 2021.11) to obtain ASVs [43]. Taxonomic assignment was performed using SILVA 138 [44] for bacteria and UNITE 8.0 [45] for fungi. Alpha diversity indices were calculated using QIIME2. Beta diversity was assessed by PCoA based on weighted UniFrac distances [46]. All detailed procedures are provided in Text S4.

### 2.5. Statistical Analysis

All experiments were conducted with three biological replicates. Data are presented as mean ± standard deviation (SD). Significant differences among treatments within the same sampling time or growth period were assessed using one-way analysis of variance (ANOVA) followed by Duncan’s multiple range test (*p* < 0.05), performed using SPSS Statistics 23.0 (IBM Corp., Armonk, NY, USA). To evaluate relationships among plant growth parameters, soil physicochemical properties, enzyme activities, lignocellulose components, and microbial community composition, three correlation methods were employed: Pearson correlation for assessing linear relationships among normally distributed continuous variables; Spearman rank correlation for evaluating monotonic relationships without assuming normality; and Kendall rank correlation, which is particularly suitable for small sample sizes and provides robust estimates in the presence of ties or outliers. The results of all three methods are presented to cross-validate the observed associations and enhance the robustness of our conclusions. Principal component analysis (PCA) was conducted using R (version 4.1.0) with the prcomp function from the stats package. All variables were centered and scaled to unit variance (scale. = TRUE) prior to PCA to ensure that variables with different units and magnitudes contributed equally to the analysis. Correlation heatmaps were generated using the corrplot package in R for all three correlation methods (Pearson, Spearman, and Kendall).

## 3. Results

### 3.1. SMS Amendment Enhances Blueberry Growth and Soil Fertility

**Figure 1:**
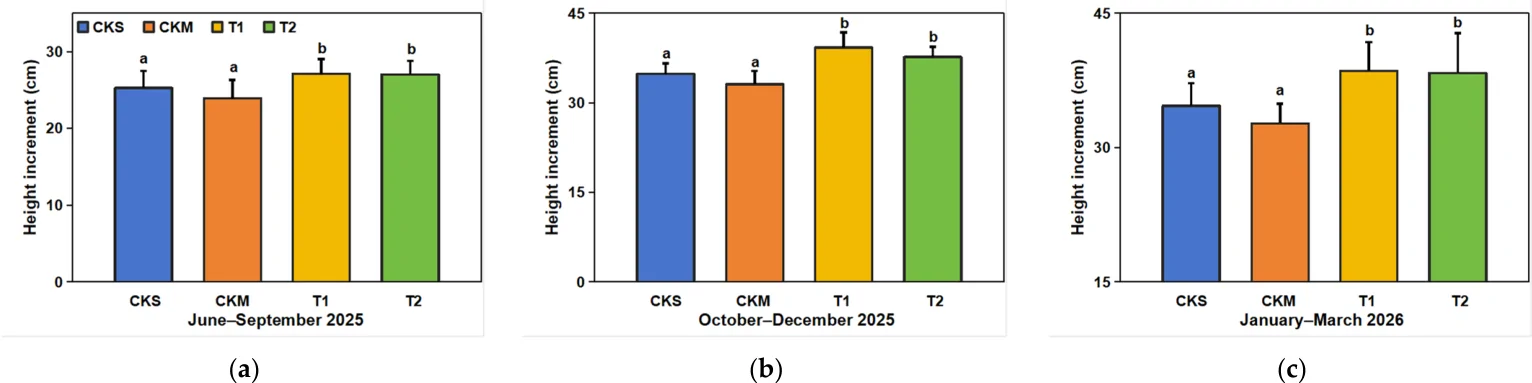
Height increment of blueberry plants under different treatments across three growth periods. (**a**) Height increment (cm) from June to September. (**b**) Height increment (cm) from October to December. (**c**) Height increment (cm) from January to March. The increment represents the net increase in shoot height during each period, calculated as the difference between height measurements at the beginning and end of each respective period. The initial height of the transplants at the zero time point (15 May 2025) was approximately 30 cm (mean across all treatments, with no significant differences among treatment groups at transplanting). Data are presented as mean ± SD (*n* = 3). Different lowercase letters above the bars indicate significant differences among treatments within the same period (*p* < 0.05, Duncan’s multiple range test). CKS: conventional cultivation soil (control); CKM: conventional soil + sterilized mushroom residue (4:1, *w*/*w*); T1: sterilized mushroom residue treated with strain T1 (54 d) mixed with conventional soil (1:4); T2: sterilized mushroom residue treated with strain T2 (54 d) mixed with conventional soil (1:4).

During the rapid growth phase (June to September 2025), plant height increments in T1 and T2 were significantly higher than those in CKS and CKM (*p* < 0.05) (Figure 1a). Growth was slowed from October to December 2025, but T1 and T2 maintained a growth advantage (Figure 1b). From January to March 2026, CKM showed slightly better performance than CKS, although the difference was not significant (Figure 1c).

**Figure 2:**
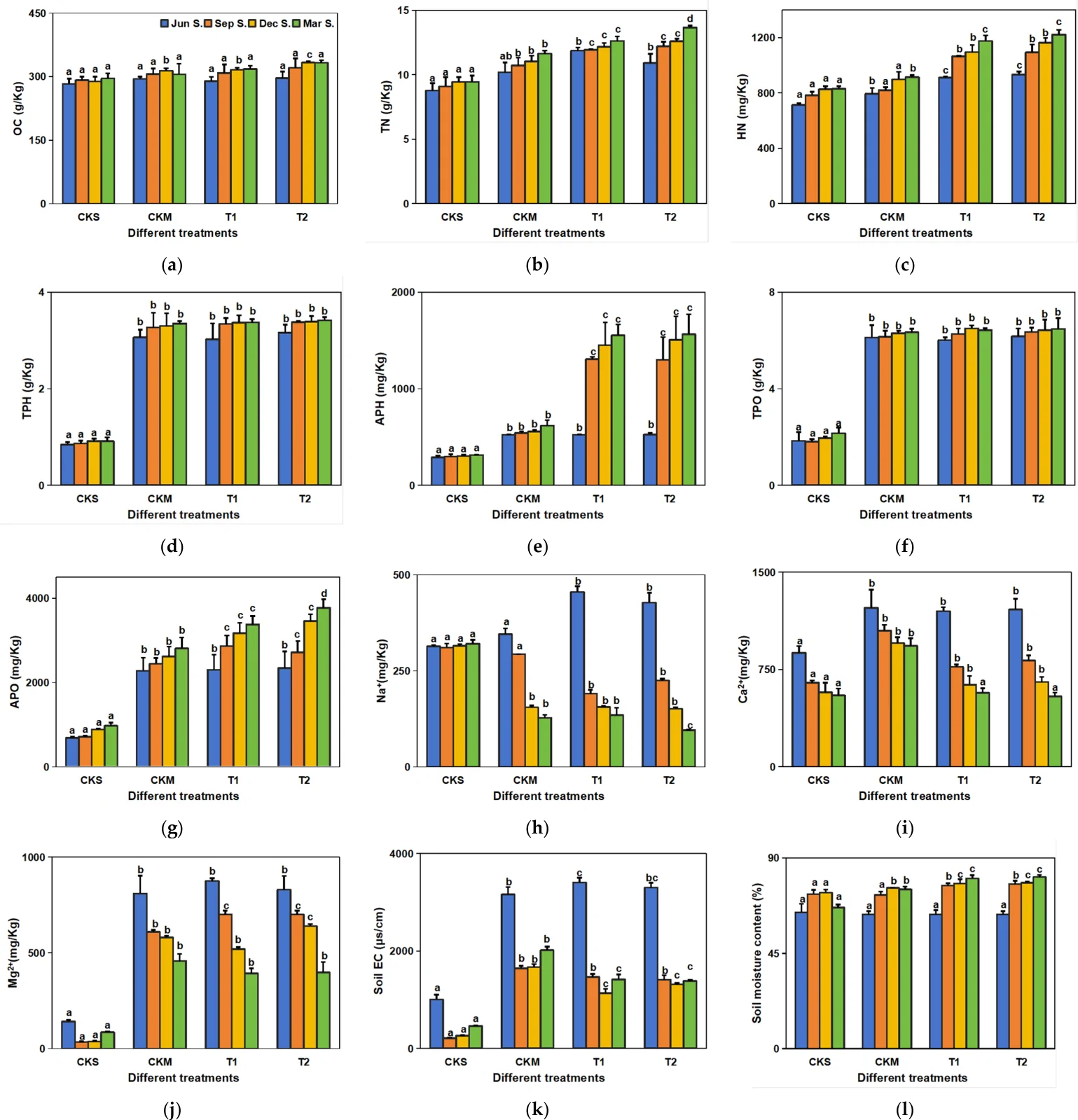
Dynamics of major nutrient elements and other physicochemical properties in blueberry rhizosphere soil under different treatments across four sampling times (June 2025, September 2025, December 2025, and March 2026). (**a**) Soil organic carbon (OC) content (g/kg) in the rhizosphere soil. (**b**) Total nitrogen (TN) content (g/kg) in the rhizosphere soil. (**c**) Alkaline hydrolysis nitrogen (HN) content (mg/kg) in the rhizosphere soil. (**d**) Total phosphorus (TPH) content (g/kg) in the rhizosphere soil. (**e**) Available phosphorus (APH) content (mg/kg) in the rhizosphere soil. (**f**) Total potassium (TPO) content (g/kg) in the rhizosphere soil. (**g**) Available potassium (APO) content (mg/kg) in the rhizosphere soil. (**h**) Sodium ion (Na^+^) content (mg/kg) in the rhizosphere soil. (**i**) Calcium ion (Ca^2+^) content (mg/kg) in the rhizosphere soil. (**j**) Magnesium ion (Mg^2+^) content (mg/kg) in the rhizosphere soil. (**k**) Soil electrical conductivity (Soil EC) (μs/cm) of the rhizosphere soil. (**l**) Soil moisture content (%) of the rhizosphere soil. Data are presented as mean ± SD (*n* = 3). Different lowercase letters above the bars indicate significant differences among treatments within the same period (*p* < 0.05, Duncan’s multiple range test). CKS: conventional cultivation soil (control); CKM: conventional soil + sterilized mushroom residue (1:4, *v*/*v*); T1: sterilized mushroom residue treated with PGPR strain T1 mixed with conventional soil (1:4, *v*/*v*); T2: sterilized mushroom residue treated with PGPR strain T2 mixed with conventional soil (1:4, *v*/*v*).

At the first sampling time (June 2025), significantly higher nutrient levels were already detected in CKM, T1, and T2 than in CKS. For instance, total phosphorus (TPH) in CKM, T1, and T2 was measured in the range of 3.07 ± 0.14 to 3.16 ± 0.16 g/kg, whereas CKS contained only 0.85 ± 0.10 g/kg (Figure 2d). Similarly, total potassium (TPO) was more than 6.01 g/kg in all SMS-added treatments, compared with 1.70 ± 0.35 g/kg in CKS (Figure 2f). Over subsequent sampling times, only minor fluctuations in organic carbon (OC, 283.00 ± 12.00 to 295.67 ± 12.50 g/kg), total nitrogen (TN, 8.99 ± 1.90 to 9.45 ± 0.48 g/kg), and available phosphorus (APH, 289.11 ± 20.03 to 314.00 ± 5.29 mg/kg) were observed in the CKS treatment (Figure 2a,b,e). In contrast, high nutrient levels were continuously increased and maintained in treatments with PGPR-treated SMS. Available potassium (APO) in T1 and T2 was increased from 2303.33 ± 356.42 and 2344.00 ± 356.44 mg/kg in June 2025 to 2866.67 ± 251.66 and 2714.00 ± 273.47 mg/kg in September 2025, and remained above 3300 mg/kg in March 2026 (Figure 2g). A marked increase in alkaline hydrolysis nitrogen (HN) was also observed in T1 and T2, from 910.00 ± 10.00 and 933.00 ± 20.66 mg/kg (June 2025) to 1176.00 ± 39.85 and 1220.67 ± 34.93 mg/kg in March 2026 (Figure 2c).

Soil electrical conductivity (EC) and the concentrations of Na^+^, Ca^2+^, and Mg^2+^ were reduced in T1 and T2 treatments compared with CKS and CKM (Figure 2h–k). Soil moisture content in T1 and T2 was consistently higher than in CKS and CKM throughout the sampling period (Figure 2l).

### 3.2. SMS Amendment Drives Enzyme Activation and Lignocellulose Degradation

**Figure 3:**
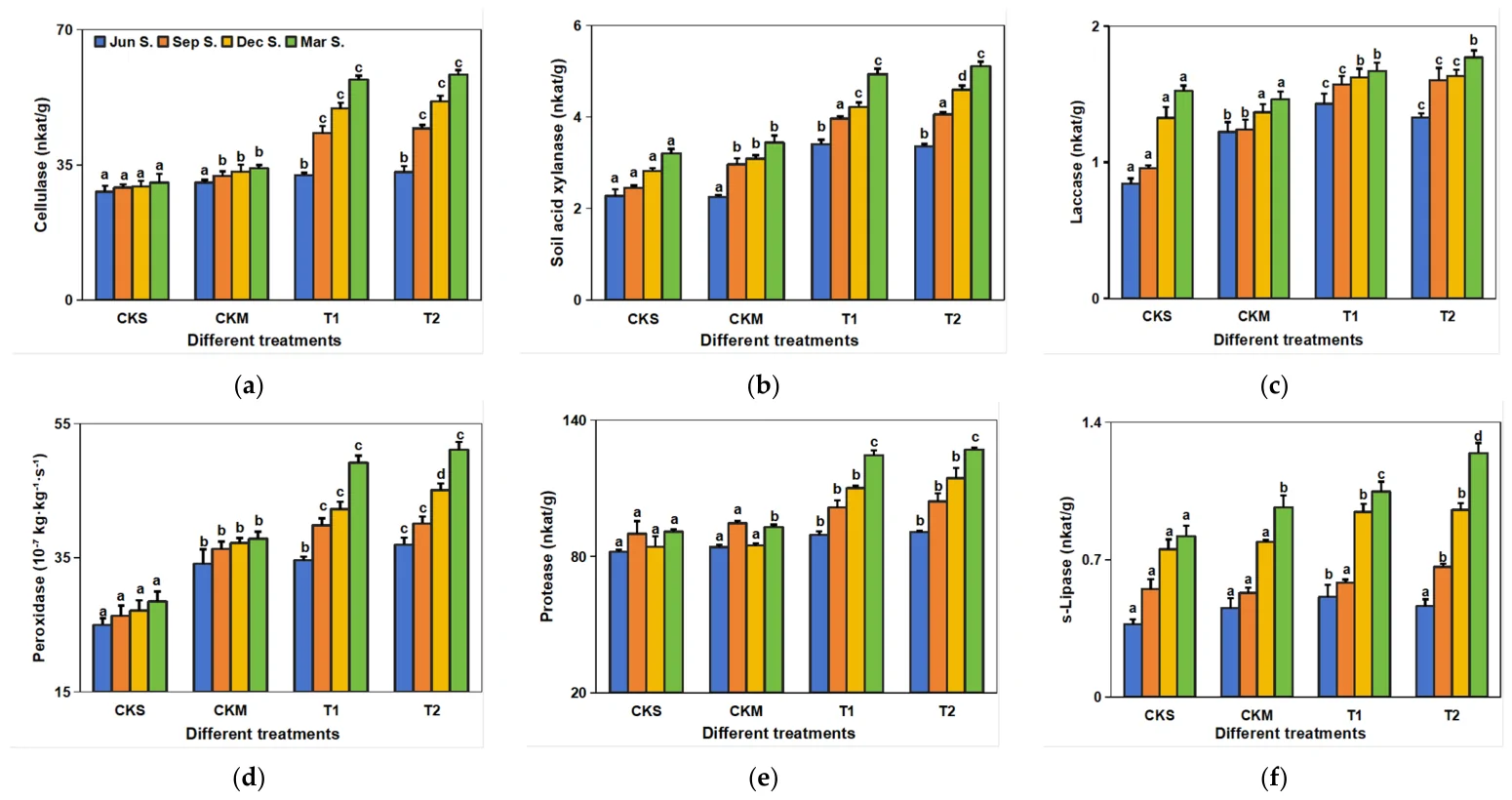

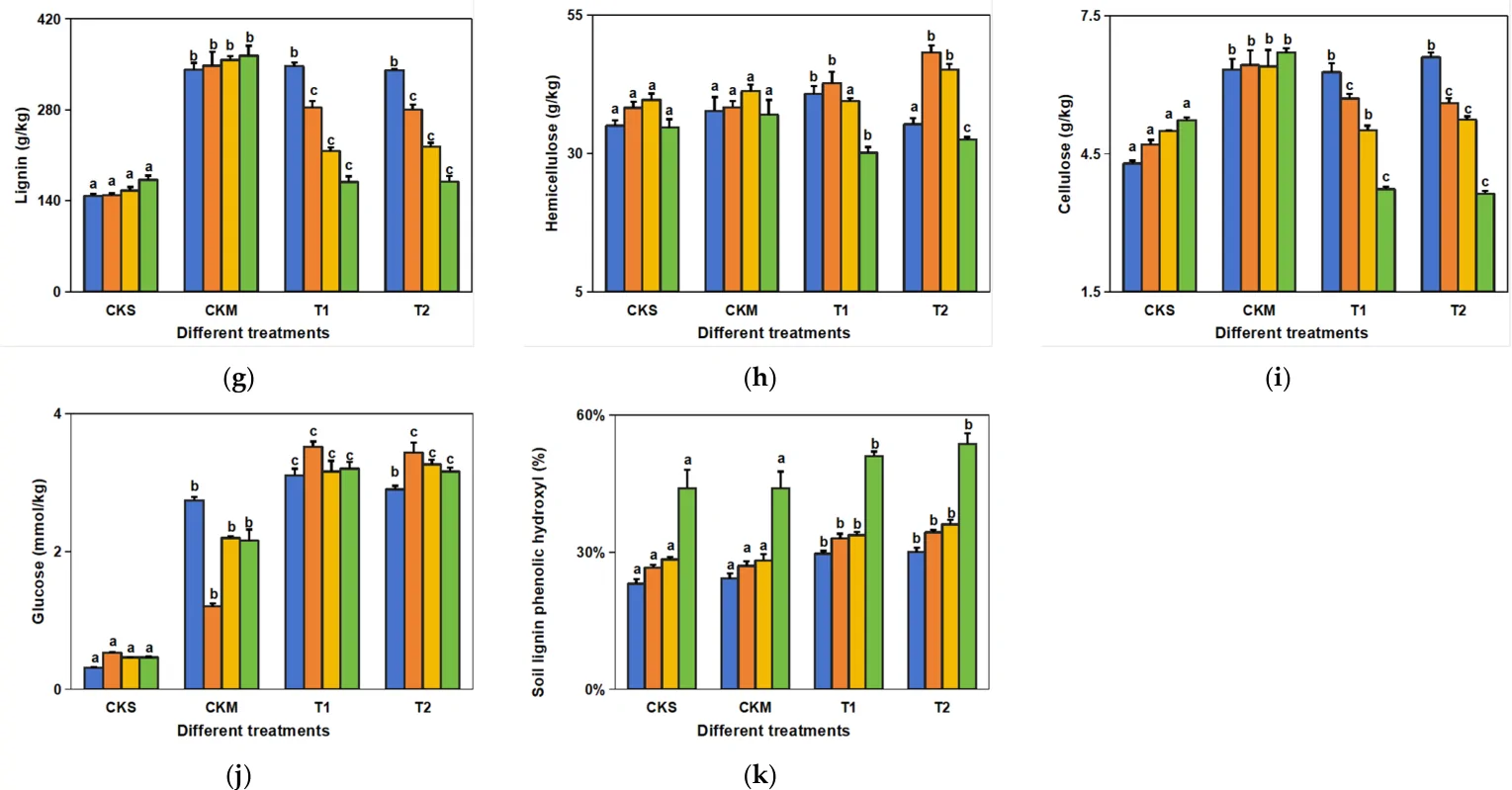
Changes in rhizosphere soil enzyme activities, lignocellulose components, and lignin phenolic hydroxyl ratio under different treatments across four sampling times (June 2025, September 2025, December 2025, and March 2026). (**a**) Cellulase activity (nkat/g) in the rhizosphere soil. (**b**) Xylanase activity (nkat/g) in the rhizosphere soil. (**c**) Laccase activity (nkat/g) in the rhizosphere soil. (**d**) Peroxidase activity (10^−7^ kg·kg^−1^·s^−1^) in the rhizosphere soil. (**e**) Protease activity (nkat/g) in the rhizosphere soil. (**f**) Lipase activity (nkat/g) in the rhizosphere soil. (**g**) Lignin content (g/kg) in the rhizosphere soil. (**h**) Hemicellulose content (g/kg) in the rhizosphere soil. (**i**) Cellulose content (g/kg) in the rhizosphere soil. (**j**) Glucose content (mmol/kg) in the rhizosphere soil. (**k**) Lignin phenolic hydroxyl ratio (%) in the rhizosphere soil. Data are presented as mean ± SD (*n* = 3). Different lowercase letters above the bars indicate significant differences among treatments within the same sampling time (*p* < 0.05, Duncan’s multiple range test). Treatment abbreviations are as defined in Figure 1.

At the initial transplanting time (June 2025), most enzyme activities were slightly increased by the addition of SMS compared with CKS, but the differences were small. Over the following months, the activities of cellulase, xylanase, laccase, peroxidase, protease, and lipase were dramatically increased in treatments with PGPR-treated SMS, peaking in March 2026 (Figure 3a–f). For example, cellulase activity was raised from 33.1 to 58.3 nkat/g in the T2 treatment; xylanase was increased from 3.36 to 5.11 nkat/g. Low and stable enzyme levels were maintained in CKS throughout the monitoring period. Moderate increases were shown in CKM, but its enzyme activities were significantly lower than those in treatments with PGPR-treated SMS (*p* < 0.05) (Figure 3a–f).

Initial lignin contents were similar in CKM and treatments with PGPR-treated SMS, much higher than in CKS. Over time, lignin in CKS remained low, while in CKM it stayed high with little degradation. In treatments with PGPR-treated SMS, lignin was sharply decreased (Figure 3g), and cellulose also declined over time (Figure 3i). Hemicellulose peaked in September 2025 and decreased in March 2026 (Figure 3h). From September onward, glucose in treatments with PGPR-treated SMS remained consistently high, while very low glucose was observed in CKS and intermediate levels were detected in CKM (Figure 3j).

The phenolic hydroxyl ratio was increased in all treatments over time, but most dramatically in treatments with PGPR-treated SMS (Figure 3k).

### 3.3. SMS Amendment Restructures Rhizosphere Microbial Communities

**Figure 4:**
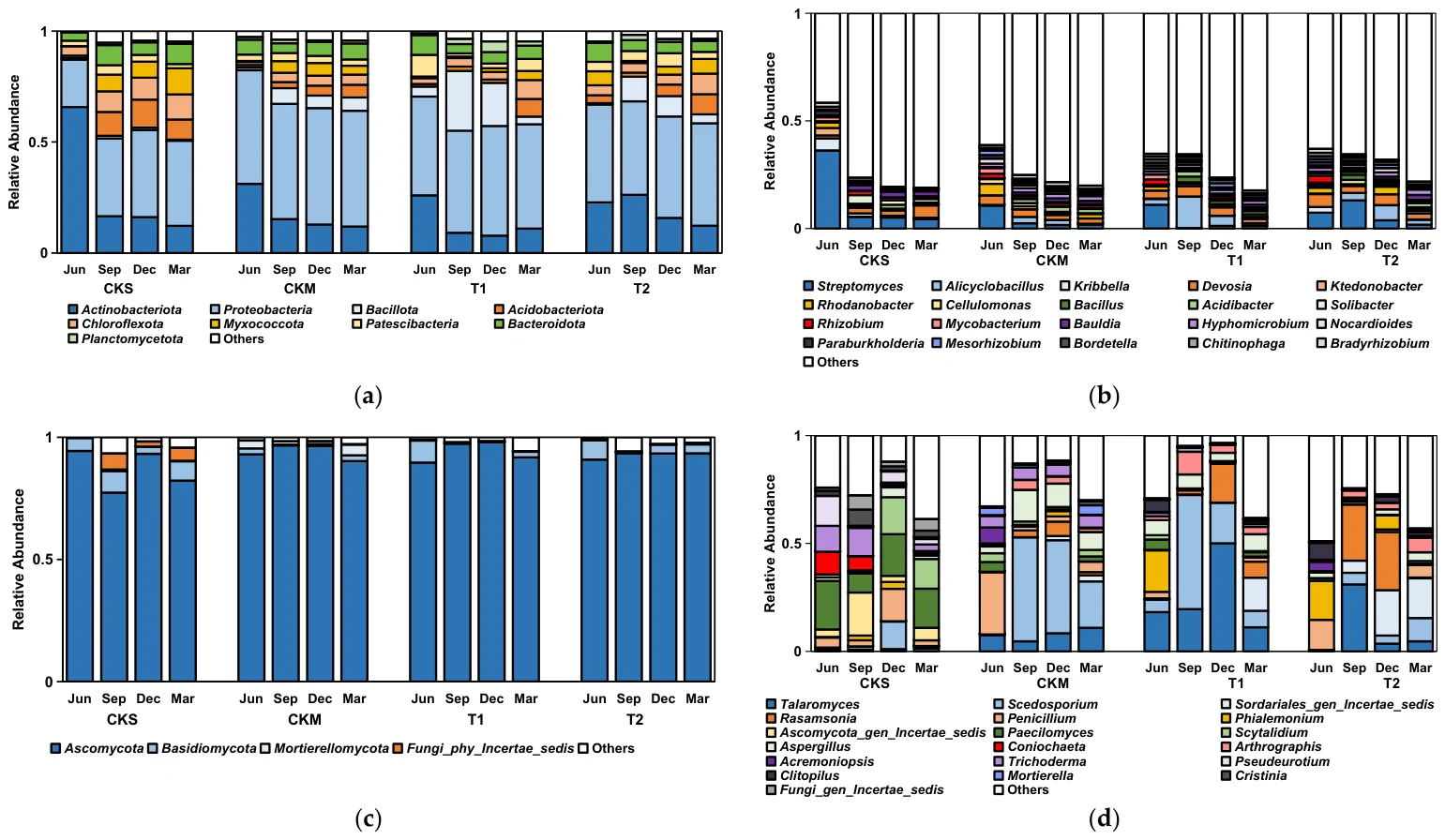
Microbial community in the blueberry rhizosphere soil under different treatments. (**a**) Composition of bacterial phyla with a relative abundance ≥ 1%, and (**b**) composition of bacterial genera. (**c**) Composition of fungal phyla and (**d**) fungal genera.

At the phylum level (Figure 4a), the bacterial communities were dominated by *Actinobacteriota*, *Bacillota*, *Acidobacteriota*, *Chloroflexota*, *Myxococcota*, and *Planctomycetota*, with phylum-level nomenclature following Oren and Garrity (2021) [47]. A higher relative abundance of *Actinobacteriota* was observed in CKS and CKM. In contrast, *Bacillota* and *Acidobacteriota* were more abundant in PGPR treatments. By Venn diagrams (Figure S2), a substantial number of unique OTUs was observed in treatments with PGPR-treated SMS, and the shared OTU numbers among treatments decreased over time.

At the genus level (Figure 4b), *Streptomyces*, *Kribbella*, and *Ktedonobacter* were more abundant in the control treatments. In treatments with PGPR-treated SMS, *Acidibacter*, *Hyphomicrobium*, and *Rhodanobacter* were enriched.

The fungal communities (Figure 4c,d) were dominated by *Basidiomycota* and *Ascomycota*. A higher relative abundance of *Basidiomycota* was found in PGPR treatments, while *Ascomycota* was more abundant in the controls. At the genus level, differential abundances among treatments were shown for *Arthrographis*, *Rasamsonia*, and *Penicillium*.

By LEfSe analysis (Figure S3), Acidibacter and Hyphomicrobium were identified as bacterial biomarkers for PGPR-treated SMS, and *Basidiomycota*-affiliated lineages were enriched as fungal biomarkers in soils amended with PGPR-treated SMS.

**Figure 5:**
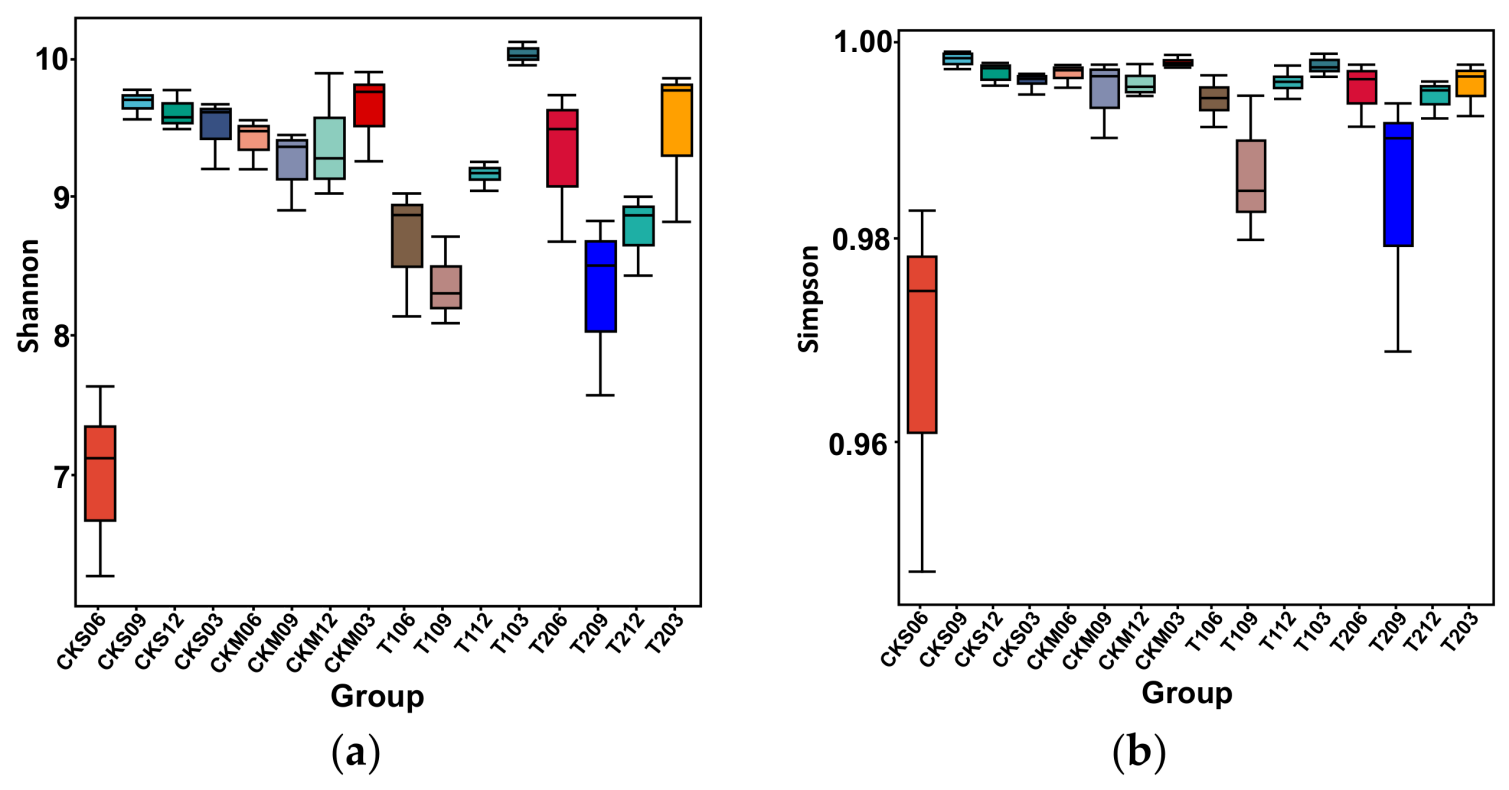

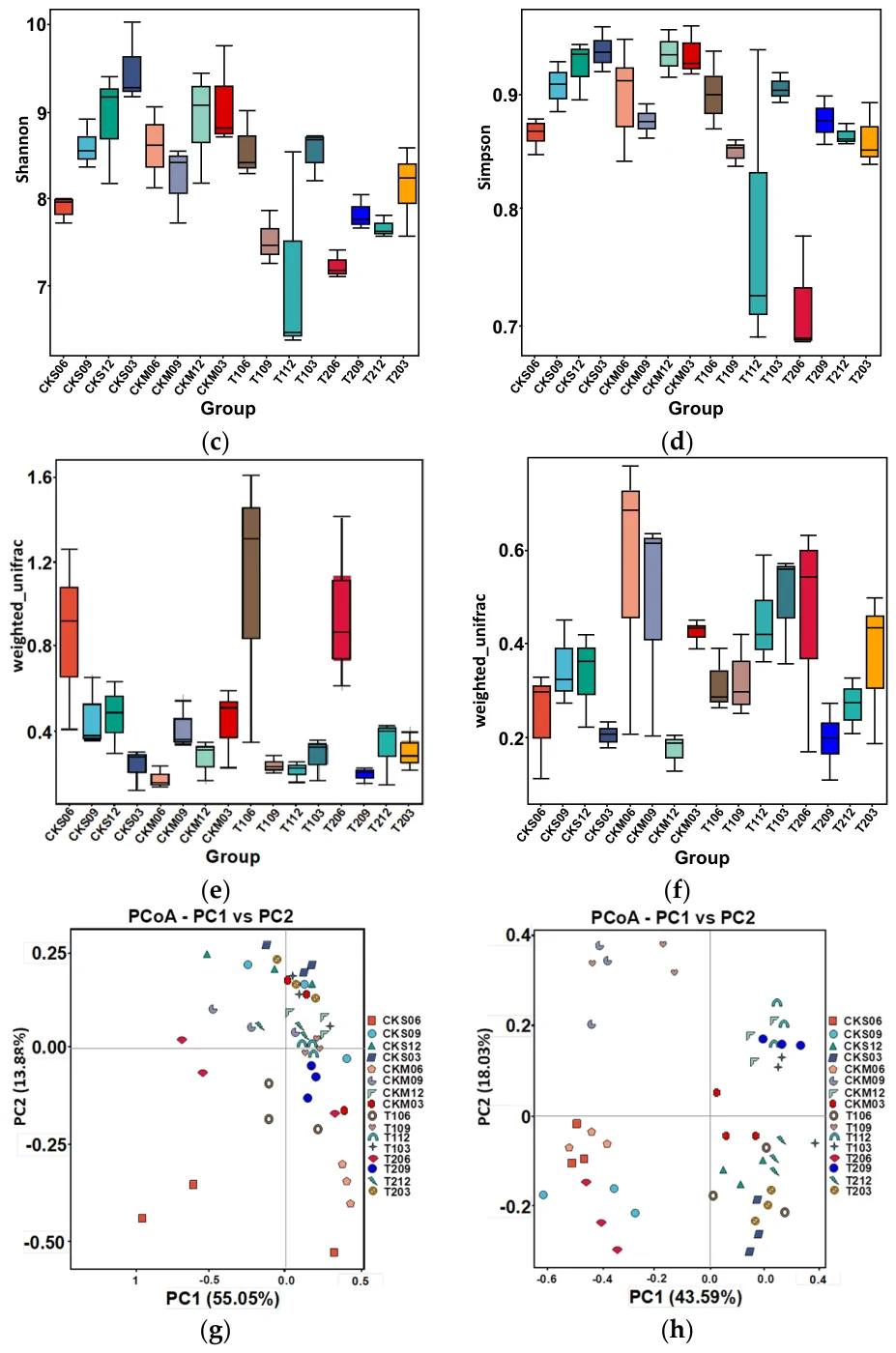
Microbial community in the blueberry rhizosphere soil under different treatments. Bacterial alpha-diversity is shown using the (**a**) Shannon index and (**b**) Simpson index, while fungal alpha-diversity is shown using the (**c**) Shannon and (**d**) Simpson indices. Phylogenetic beta-diversity based on weighted UNIFRAC distances is presented for (**e**) bacterial and (**f**) fungal communities. Finally, (**g**,**h**) Principal coordinates analysis (PCoA) plot based on weighted UniFrac distances for bacterial and fungal communities, respectively.

Bacterial alpha diversity, measured by Shannon and Simpson indices (Figure 5a,b), was significantly higher in treatments with PGPR-treated SMS than in CKS and CKM. A similar trend was shown for fungal alpha diversity (Figure 5c,d). By principal coordinate analysis (PCoA) based on weighted UniFrac distances (Figure 5g,h), clear separation among treatments was observed. PC1 explained 43.59% of the variance, and PC2 explained 18.80% of the variance, together accounting for 62.39% of the total variation. Samples from treatments with PGPR-treated SMS were clustered distinctly from CKS and CKM.

### 3.4. Integrated Correlation and PCA Validate Soil Fertility Enhancement

**Figure 6:**
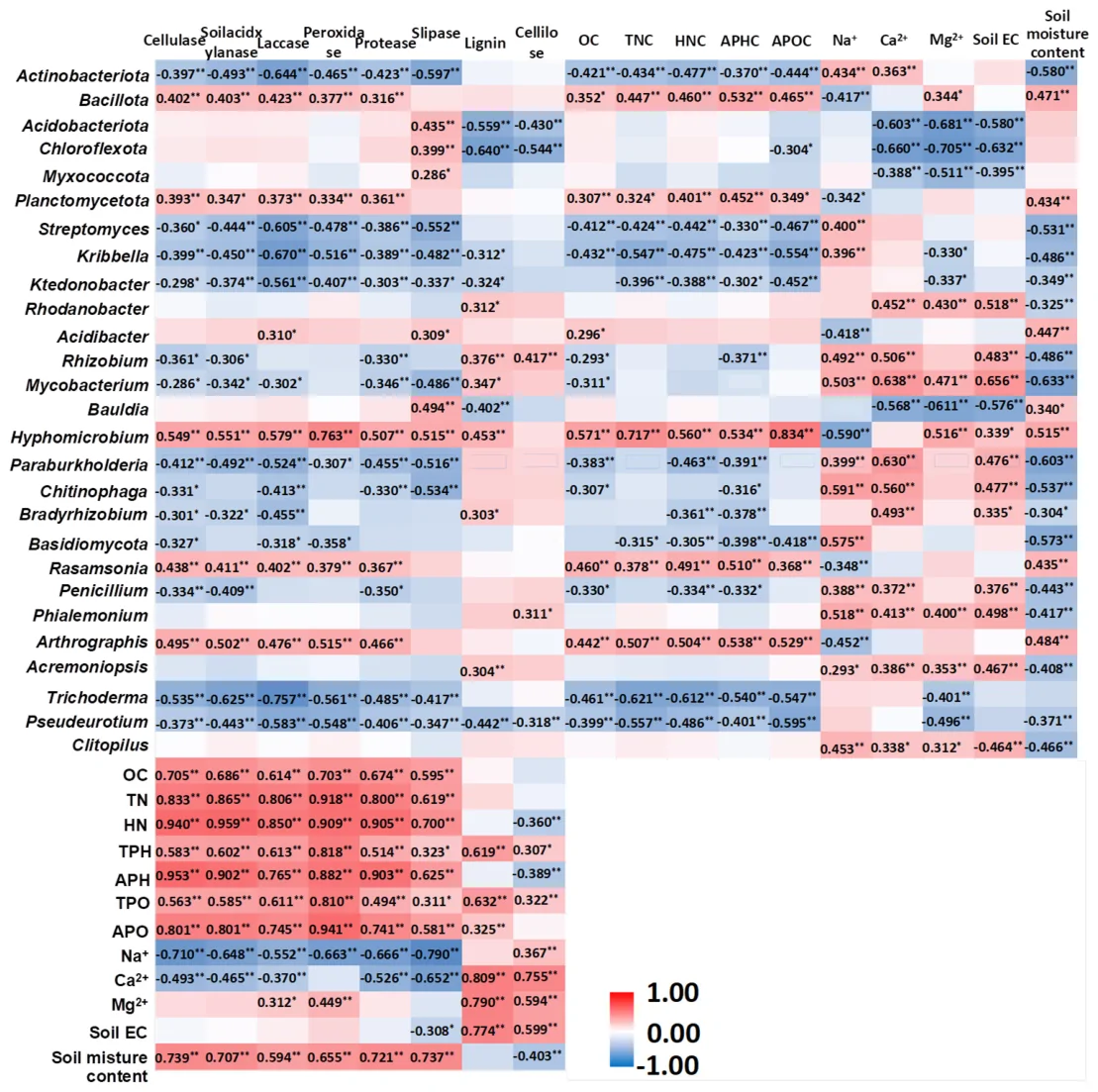
Integrated correlation heatmap showing associations among rhizosphere microbial community structure, nutrient elements, soil enzyme activities, lignocellulose components, and lignin phenolic hydroxyl ratio, and other physicochemical properties in blueberry rhizosphere soil. The color scale represents the Pearson correlation coefficient, ranging from −1 (negative correlation, blue) to +1 (positive correlation, red). Data are presented from three biological replicates (*n* = 3). * and ** indicate significant correlations at *p* < 0.05 and *p* < 0.01, respectively (based on two-tailed *t*-tests). Note: OC: Soil organic carbon, TN: Total nitrogen, HN: Alkaline hydrolysis nitrogen, TPH: Total phosphorus, APH: Available phosphorus, TPO: Total potassium, APO: Available potassium, Na^+^: Sodium ion, Ca^2+^: Calcium ion, Mg^2+^: Magnesium ion, Soil EC: Soil electrical conductivity.

From the integrated correlation heatmap (Figure 6) and detailed Pearson analysis (Table 1), the height increment of blueberry plants was found to be positively correlated with soil organic carbon (OC, *r* = 0.533, *p* < 0.01), total nitrogen (TN, *r* = 0.382, *p* < 0.01), alkaline hydrolysis nitrogen (HN, *r* = 0.542, *p* < 0.01), available phosphorus (APH, *r* = 0.512, *p* < 0.01), available potassium (APO, *r* = 0.354, *p* < 0.05), and all six measured enzyme activities—cellulase (*r* = 0.567, *p* < 0.01), xylanase (*r* = 0.592, *p* < 0.01), laccase (*r* = 0.598, *p* < 0.01), peroxidase (*r* = 0.449, *p* < 0.01), protease (*r* = 0.582, *p* < 0.01), and lipase (recorded as Lipase, *r* = 0.824, *p* < 0.01), as well as with soil moisture content (*r* = 0.850, *p* < 0.01). In terms of microbial taxa, positive associations were observed with the phyla *Bacillota* (*r* = 0.309, *p* < 0.05), *Acidobacteriota* (*r* = 0.510, *p* < 0.01), *Chloroflexota* (*r* = 0.482, *p* < 0.01), *Myxococcota* (*r* = 0.376, *p* < 0.01), and *Planctomycetota* (*r* = 0.342, *p* < 0.05), and with the genera *Acidibacter* (*r* = 0.417, *p* < 0.01), *Bauldia* (*r* = 0.605, *p* < 0.01), *Hyphomicrobium* (*r* = 0.337, *p* < 0.05), as well as the fungal genera *Arthrographis* (*r* = 0.320, *p* < 0.05) and *Rasamsonia* (*r* = 0.325, *p* < 0.05). Conversely, negative correlations were detected with lignin (*r* = –0.364, *p* < 0.05), cellulose (*r* = –0.381, *p* < 0.01), sodium (Na^+^, *r* = –0.793, *p* < 0.01), calcium (Ca^2+^, *r* = –0.794, *p* < 0.01), magnesium (Mg^2+^, *r* = –0.373, *p* < 0.01), soil electrical conductivity (EC, *r* = –0.651, *p* < 0.01), and with the bacterial phylum *Actinobacteriota* (*r* = –0.702, *p* < 0.01), as well as the genera *Streptomyces* (*r* = –0.621, *p* < 0.01), *Kribbella* (*r* = –0.536, *p* < 0.01), *Ktedonobacter* (*r* = –0.367, *p* < 0.05), *Rhodanobacter* (*r* = –0.479, *p* < 0.01), *Rhizobium* (*r* = –0.426, *p* < 0.01), *Mycobacterium* (*r* = –0.780, *p* < 0.01), *Paraburkholderia* (*r* = –0.723, *p* < 0.01), *Chitinophaga* (*r* = –0.702, *p* < 0.01), and *Bradyrhizobium* (*r* = –0.405, *p* < 0.01). Additionally, negative correlations were shown for the fungal phylum *Basidiomycota* (*r* = –0.379, *p* < 0.01) and the genera *Penicillium* (*r* = –0.451, *p* < 0.01), *Phialemonium* (*r* = –0.440, *p* < 0.01), *Acremoniopsis* (*r* = –0.435, *p* < 0.01), *Trichoderma* (*r* = –0.287, *p* < 0.05), *Pseudeurotium* (*r* = –0.330, *p* < 0.05), and *Clitopilus* (*r* = –0.458, *p* < 0.01). Consistent patterns were also confirmed by Kendall and Spearman correlation analyses (Table S1 and Table S2, Figure S4).

Principal component analysis (Table S3) revealed that PC1, which accounted for 43.59% of the total variance, was characterized by high positive loadings for all six enzyme activities (0.821–0.900), OC (0.744), TN (0.805), HN (0.891), APH (0.864), APO (0.776), and soil moisture content (0.878), while strong negative loadings were observed for *Actinobacteriota* (–0.709), *Streptomyces* (–0.660), *Kribbella* (–0.660), Na^+^ (–0.806), and Ca^2+^ (–0.557). PC2, explaining 18.80% of the variance, exhibited high positive loadings for lignin (0.782), cellulose (0.582), Ca^2+^ (0.739), Mg^2+^ (0.948), and soil EC (0.887), together with high negative loadings for *Acidobacteriota* (–0.783), *Chloroflexota* (–0.823), *Myxococcota* (–0.639), and *Bauldia* (–0.722). These results collectively indicate that the PGPR-treated SMS amendment primarily enhanced soil fertility and enzymatic activity (PC1), while simultaneously reducing recalcitrant lignocellulose components and salt accumulation (PC2), thereby providing a mechanistic basis for the observed improvement in plant growth.

## 4. Discussion

### 4.1. SMS Amendment Promotes Blueberry Growth and Sustains Soil Fertility

The growth-promoting effect of SMS was significantly enhanced by cultivation with functional strains T1 and T2. At the first sampling time, significantly higher nutrient levels were already detected in CKM, T1, and T2 than in CKS, because abundant organic carbon, nitrogen, phosphorus, and potassium were introduced by SMS addition [48,49]. The persistently elevated nutrient levels in treatments with PGPR-treated SMS suggest that the decomposition of the added organic matter continuously released nutrients that would otherwise remain bound in the SMS matrix or soil organic fractions. The cultivation of SMS with PGPR is known to enhance the production of organic acids and phosphatases that solubilize insoluble phosphates and release potassium from organic complexes [20,50]. This process also accelerates the mineralization of organic nitrogen, which explains the rising HN observed in treatments with PGPR-treated SMS [51]. The lower EC in soils amended with PGPR-treated SMS alleviates osmotic stress, because high salt concentrations can impair root water uptake and cause ion toxicity [52,53]. The consistently higher soil moisture content in T1 and T2 may be attributed to improved soil structure and water-holding capacity resulting from organic matter addition [54]. In summary, PGPR-treated SMS acted as multifunctional organic amendment that not only supplied nutrients upfront but also sustained their availability over at least ten months, primarily through microbial mobilization of recalcitrant nutrient pools, providing a solid mechanistic basis for the enhanced plant growth. It should be noted that the substrate pH was within the range suitable for blueberry cultivation (4.5–5.5), as the peat-based nutrient soil used had a specified pH of 5.5–6.0. Although pH can influence enzyme stability and microbial community composition [55,56], the treatment-specific differences observed here are primarily attributable to the SMS amendments and PGPR inoculation, as all treatments were established under the same initial substrate conditions.

### 4.2. Enzyme Activation and Lignocellulose Degradation Driven by SMS Amendment

The marked increases in cellulase, xylanase, laccase, peroxidase, protease, and lipase activities provide direct evidence for nutrient release. Cellulase and xylanase are key enzymes for the breakdown of cellulose and hemicellulose [57]; laccase and peroxidase are essential for lignin degradation by breaking aromatic rings [58]. The increases in protease and lipase indicate accelerated decomposition of organic matter [59]. The cultivation of SMS with functional strains acted as a trigger, activating the soil microbial community to produce more extracellular enzymes. The sharp decrease in lignin in PGPR-treated treatments matches the increases in laccase and peroxidase activities. Cellulose decline is a direct result of elevated cellulase activity. The initial increase in hemicellulose may be explained by the decomposition of other easily accessible components, making hemicellulose relatively more concentrated; later, as xylanase activity rose, hemicellulose itself was hydrolyzed [60,61]. The sustained high glucose in PGPR-treated treatments was regarded as a feedback signal that further stimulated microbial activity and proliferation, establishing a positive feedback mechanism [62]. The large increase in the phenolic hydroxyl ratio indicates that lignin underwent extensive oxidative modification—one-electron oxidation reactions catalyzed by laccase and peroxidase attack phenolic hydroxyl groups and side chains, causing Cα–Cβ bond cleavage, aromatic ring opening, and demethylation [63]. This makes lignin looser, more hydrophilic, and susceptible to further hydrolysis and mineralization, releasing trapped cellulose and hemicellulose. Together, these findings reveal a cascade of nutrient mobilization, where PGPR-treated SMS stimulates hydrolase and oxidoreductase activities, depolymerizing lignocellulose and releasing locked nutrients, thus creating a fertile, actively cycling rhizosphere.

### 4.3. Microbial Community Restructuring Under SMS Amendment

The enrichment of *Bacillota* and *Acidobacteriota* in PGPR treatments is functionally significant: *Bacillota* are key players in the decomposition of organic polymers [64], and *Acidobacteriota* influence rhizosphere biogeochemical cycles, including nitrogen, phosphorus, and plant growth dynamics [65]. Their enrichment aligns with the enhanced lignocellulose degradation and nutrient cycling observed. The presence of unique OTUs in PGPR-treated treatments indicates that specific bacterial taxa were introduced or selected by the SMS amendment. At the genus level, enrichment of *Acidibacter*—associated with phosphorus solubilization [66]—may explain the increased available phosphorus; enrichment of *Hyphomicrobium*—which participates in nitrogen cycling [67]—may contribute to sustained nitrogen availability. It is noteworthy that the inoculated PGPR strains (*Pseudomonas* sp. T1 and *Buttiauxella* sp. T2) were not detected as dominant genera in the rhizosphere at the sampling time points (Figure 4a,b). This observation is consistent with previous reports showing that PGPR can exert growth-promoting effects by modulating the resident microbial community structure rather than by maintaining high population densities [68,69,70]. For instance, Jiang et al. [68] demonstrated that even when PGPRs fail to colonize natural soil, they can still decrease niche breadth and increase niche overlap by 59.2–62.4%, thereby reshaping the resident microbial community structure through intensified competition. Similarly, Mallon et al. [69] showed that PGPR introductions that do not persist in the community may still leave legacy effects by altering the abundance of key functional groups or changing interaction networks. Furthermore, Larcher et al. [70] highlighted that the efficacy of a PGPR is not primarily dependent on the extent of its root colonization. In the present study, the two PGPR strains were applied during the pre-treatment phase (54 days of aerobic decomposition) prior to transplanting. By the time of the first rhizosphere sampling (June 2025), the inoculated strains had already fulfilled their role as “initiators”—triggering the decomposition of lignocellulose and the release of nutrients—rather than persisting as long-term dominant colonizers. The subsequent enrichment of functional taxa such as *Bacillota*, *Acidobacteriota*, *Acidibacter*, and *Hyphomicrobium* in T1 and T2 treatments (as shown in Figure 4) represents the legacy effect of the PGPR inoculation: the initial microbial stimulation reshaped the rhizosphere environment, which in turn selected for indigenous taxa that sustain enzyme activities, lignocellulose degradation, and nutrient cycling over the ten-month experimental period. The Shannon and Simpson indices consistently showed significantly higher bacterial and fungal diversity in T1 and T2 treatments compared to CKS and CKM. This increase in α-diversity indicates that PGPR-treated SMS created a more heterogeneous and nutrient-rich rhizosphere environment, supporting a wider array of microbial taxa [71]. The shift toward *Basidiomycota* dominance in PGPR treatments is meaningful because various orders within *Basidiomycota* are known to play important roles in lignocellulose degradation [72], consistent with enhanced lignin degradation and elevated laccase/peroxidase activities. The identification of *Acidibacter*, *Hyphomicrobium*, and *Basidiomycota*-affiliated lineages as biomarkers through LEfSe further confirms that SMS amendment selected for specific microbial taxa supporting enzyme activities and nutrient cycling.

Higher microbial diversity, as observed in PGPR-treated treatments, is generally associated with more stable and functional ecosystems and may reflect the creation of a more heterogeneous and nutrient-rich environment [73]. The clear separation of samples in PCoA, with PC1 primarily associated with differences in soil nutrient contents and enzyme activities, and PC2 separating based on microbial community composition, confirms that PGPR-treated SMS fundamentally reshaped the rhizosphere microbiome. These results are consistent with previous findings that PGPR-treated organic amendments can significantly modify rhizosphere microbial community structure [74]. The coordinated microbial response—from overall community structure and alpha diversity down to specific taxa—provides a mechanistic link between enhanced enzyme activities, lignocellulose degradation, and sustained plant growth and soil fertility.

### 4.4. Integrated Correlation and PCA Confirm Soil Fertility Enhancement by SMS Amendment

The positive correlations between plant height and soil nutrients confirm nutrient availability as a primary driver of blueberry growth. Among enzyme activities, lipase showed the highest correlation, followed by laccase and xylanase, suggesting that lipid and lignocellulose decomposition played important roles. The positive correlation with soil moisture content indicates that improved water retention contributed to enhanced performance. Negative correlations with lignin, cellulose, and salt ions (Na^+^, Ca^2+^, Mg^2+^, EC) further support that decomposition of recalcitrant components and alleviation of salt stress are beneficial. The correlation patterns at the microbial level are consistent with the enrichment of specific functional taxa in SMS-amended soils. PCA interpretation—PC1 as the gradient of soil fertility and enzyme activities enhanced by SMS amendment, and PC2 as the gradient of lignocellulose degradation and cation accumulation—confirms that the primary axis of variation was driven by SMS addition, which enhanced soil fertility and microbial activity while reducing salt accumulation. Overall, the integrated analyses demonstrate that PGPR-treated SMS amendment enhances soil quality through multiple interconnected pathways: (1) direct nutrient supply from the organic matrix; (2) stimulation of extracellular enzyme production; (3) restructuring of the rhizosphere microbiome toward functional taxa; and (4) improvement of soil physical properties such as water-holding capacity. These findings support the conclusion that PGPR-treated SMS serves as an effective strategy for improving soil fertility and promoting plant growth in blueberry cultivation.

## 5. Conclusions

This study explored the effects of PGPR-treated spent mushroom substrate (SMS) on blueberry growth and rhizosphere soil properties, yielding key integrated findings: PGPR-treated SMS significantly promoted blueberry growth, with a more prominent facilitative effect during the rapid active growth phase from June to September compared with the slower growth period from October to March. This treatment effectively sustained soil fertility by continuously releasing and maintaining high levels of soil organic carbon, total nitrogen, alkaline hydrolysis nitrogen, available phosphorus, and available potassium throughout the experiment, in contrast to the decreased nutrient levels observed in control groups. Additionally, PGPR-modified SMS markedly enhanced the activities of multiple soil enzymes including cellulase, xylanase, laccase, peroxidase, protease, and lipase, which facilitated the degradation of soil lignin and cellulose, sustained high glucose release, and increased the phenolic hydroxyl ratio to achieve oxidative modification of lignin structure. Moreover, SMS amendment substantially restructured the rhizosphere microbial community, enriching the phyla *Bacillota* and *Acidobacteriota* and the genera *Acidibacter* and *Hyphomicrobium*, shifting the fungal community toward *Basidiomycota* dominance, and improving microbial alpha diversity, while principal coordinate analysis verified significant differences in microbial community structure between treated and control groups. Correlation and principal component analyses further confirmed strong positive correlations between blueberry growth performance, soil nutrient availability, soil enzyme activities, and rhizosphere microbial community composition. Collectively, these results demonstrate that PGPR-treated SMS serves as an efficient multifunctional soil amendment that improves soil quality and plant growth via multiple synergistic pathways including direct nutrient supplementation, enzymatic activity stimulation, lignocellulose degradation, and rhizosphere microbiome regulation. This sustainable mushroom waste recycling strategy shows substantial application potential in blueberry cultivation and can be further extended to other horticultural crops to advance eco-friendly agricultural production.

## Figures and Tables

**Table 1:** Pearson integrated correlation among Height increment of blueberry plants compared with rhizosphere microbial community structure, nutrient elements, soil enzyme activities, lignocellulose components, and lignin phenolic hydroxyl ratio, and other physicochemical properties in blueberry rhizosphere soil.

	*Actinobacteriota*	*Bacillota*	*Acidobacteriota*	*Chloroflexota*	*Myxococcota*	*Planctomycetota*	*Streptomyces*	*Kribbella*	*Ktedonobacter*	*Rhodanobacter*
Height increment	−0.702 **	0.309 *	0.510 **	0.482 **	0.376 **	0.342 *	−0.621 **	−0.536 **	−0.367 *	−0.479 **
	*Acidibacter*	*Rhizobium*	*Mycobacterium*	*Bauldia*	*Hyphomicrobium*	*Paraburkholderia*	*Chitinophaga*	*Bradyrhizobium*	-	*-*
Height increment	0.417 **	−0.426 **	−0.780 **	0.605 **	0.337 *	−0.723 **	−0.702 **	−0.405 **	-	-
	*Basidiomycota*	*Arthrographis*	*Rasamsonia*	*Penicillium*	*Phialemonium*	*Acremoniopsis*	*Trichoderma*	*Pseudeurotium*	*Clitopilus*	
Height increment	−0.379 **	0.320 *	0.325 *	−0.451 **	−0.440 **	−0.435 **	−0.287 *	−0.330 *	−0.458 **	
	Cellulase	Xylanase	Laccase	Peroxidase	Protease	Lipase	Lignin	Cellulose	-	
Height increment	0.567 **	0.592 **	0.598 **	0.449 **	0.582 **	0.824 **	−0.364 *	−0.381 **	-	-
	OC	TN	HN	APH	APO	Na^+^	Ca^2+^	Mg^2+^	Soil EC	Soil moisture content
Height increment	0.533 **	0.382 **	0.542 **	0.512 **	0.354 *	−0.793 **	−0.794 **	−0.373 **	−0.651 **	0.850 **

Note: * *p* < 0.05; ** *p* < 0.01.

## Data Availability

The data presented in this study are available in NCBI Sequence Read Archive (SRA) at PRJNA1494821.

## Supplementary Materials

The following supporting information can be downloaded at: https://www.mdpi.com/article/10.3390/microorganisms14081827/s1, Text S1. PGPR Strain Preparation and SMS Treatment; Text S2. Sample collection and determination of nutritional elements in rhizosphere soil; Text S3. Determination of enzyme activities and biochemical components in rhizosphere soil; Text S4. Soil microbial community analysis; Table S1. Kendall integrated correlation among Height increment of blueberry plants compared with rhizosphere microbial community structure, nutrient elements, soil enzyme activities, lignocellulose components, and lignin phenolic hydroxyl ratio, and other physicochemical properties in blueberry rhizosphere soil; Table S2. Spearman integrated correlation among Height increment of blueberry plants compared with rhizosphere microbial community structure, nutrient elements, soil enzyme activities, lignocellulose components, and lignin phenolic hydroxyl ratio, and other physicochemical properties in blueberry rhizosphere soil; Table S3. Eigenvectors (factor loadings) of the measured soil properties, enzyme activities, lignocellulose components, and microbial taxa on the first eight principal components (PC1–PC8) derived from principal component analysis (PCA); Figure S1. Venn diagram showing the distribution of unique and shared OTUs of the microbial community in the blueberry rhizosphere soil at four sampling times under different treatments (CKS, CKM, T1, and T2) based on high-throughput sequencing; Figure S2. LEfSe evolutionary branch diagram of the microbial community in the blueberry rhizosphere soil at four sampling times under different treatments (CKS, CKM, T1, and T2) based on high-throughput sequencing; Figure S3. Integrated correlation heatmap showing associations among rhizosphere microbial community structure, nutrient elements, soil enzyme activities, lignocellulose components, and lignin phenolic hydroxyl ratio, and other physicochemical properties in blueberry rhizosphere soil. Figure S4: Integrated correlation heatmap showing associations among rhizosphere microbial community structure, nutrient elements, soil enzyme activities, lignocellulose components, and lignin phenolic hydroxyl ratio, and other physicochemical properties in blueberry rhizosphere soil.

## Abbreviations

The following abbreviations are used in this manuscript:

SMS	Spent mushroom substrate
PGPR	Plant growth-promoting rhizobacteria
OC	Organic carbon
TN	Total nitrogen
HN	Hydrolysis nitrogen
TPH	Total phosphorus
APH	Available phosphorus
TPO	Total potassium
APO	Available potassium
EC	Electrical conductivity
