# Supplementary material for: PGPR-Treated Spent Mushroom Substrate Enhances Lignocellulose Degradation, Enzyme Activities, and Microbial Restructuring to Sustain Blueberry Rhizosphere Fertility
Source: Microorganisms. 2026 Aug 18;14(8):1827. doi: 10.3390/microorganisms14081827 (PMC13515782; doi:10.3390/microorganisms14081827)
Supplement: Supplementary file 1 [file microorganisms-14-01827-s001.zip › microorganisms-4480283-supplementary.pdf]

Article

PGPR-Treated Spent Mushroom Substrate Enhances Lignocellulose Degradation, Enzyme Activities, and Microbial Restructuring to Sustain Blueberry Rhizosphere Fertility

Mengjiao Wang [1,2,3,*], Ningqiang Li [1,2,3], Yinku Liang [1,2,3], Zhimin Xu [4] and Haicui Wu [5]

1 School of Biological Science and Engineering, Shaanxi University of Technology, Hanzhong 723001, China
2 State Key Laboratory of Qinba Resource and Ecological Environment Jointly Established by the Ministry and Ministry of Education (Cultivation), Shaanxi University of Technology, Hanzhong 723001, China
3 Shaanxi Key Laboratory of Bioresources, Shaanxi University of Technology, Hanzhong 723000, China
4 School of Nutrition and Food Sciences, Louisiana State University, Baton Rouge, LA 70802, USA
5 Department of Food Science and Nutrition, The Hong Kong Polytechnic University, Hung Hom, Kowloon, Hong Kong, China
* Correspondence: amy133253@126.com; Tel.: +86-182-9169-9362

Text S1. PGPR Strain Preparation and SMS Treatment

The SMS was air-dried naturally and ground into a fine powder using a Retsch Grindomix GM 200 at 200 rpm for 10 min. The resulting powder was then sieved through a 20-mesh sieve (0.85 mm). Two PGPR strains (designated T1 and T2) with high plant growth-promoting potential were selected based on their ability to grow effectively in SMS extract, following the methods described by Wang et al. [16]. The strains were first incubated in liquid beef extract peptone medium at 28 °C for 72 h. Sterilized SMS, PGPR culture, and sterile water were then mixed at a ratio of 1:3:10 (w:v:v) in 5 L sterile Erlenmeyer flasks and incubated at 28 °C for 72 h. The moisture content of the resulting mixture was adjusted to approximately 65% (w/w) by adding sterile distilled water, which is optimal for microbial decomposition of lignocellulosic substrates as established in our previous study [16]. The mixture was subsequently transferred to a well-ventilated environment at room temperature (25 ± 2 °C, relative humidity 60 ± 5%) for aerobic decomposition.

The PGPR-treated SMS was harvested after 54 days of decomposition. The two bacterial strains (T1 and T2) were used to prepare two corresponding PGPR-treated SMS substrates. Each treated SMS was then mixed with nutrient soil (Brighten 2024, a commercial peat-based substrate with pH 5.5-6.0, Pindstrup Group, Ryomgård, Denmark) at a ratio of 1:4 (SMS : nutrient soil, v/v) to produce the cultivation substrates for treatments T1 and T2. For the control treatments, nutrient soil alone (designated CKS) and sterilized SMS (without PGPR inoculation) mixed with the same nutrient soil at the same 1:4 ratio (designated CKM) were prepared. All substrates were thoroughly homogenised before use.

The experiment was conducted in a greenhouse using a raised-bed system to ensure adequate drainage and prevent waterlogging. Each plot measured 3 m × 2 m (6 m^2). The beds were constructed by excavating the topsoil to a depth of 20 cm and then refilling with the respective substrate to a total height of 40 cm (20 cm below and 20 cm above the original ground surface), corresponding to a substrate volume of approximately 2.4 m^3 per plot, which equates to approximately 0.8 m^3 of substrate per plant (3 plants per plot). The four treatments (T1, T2, CKS, and CKM) were arranged in a randomized complete block design with three replicate plots per treatment, giving a total of 12 experimental plots. Accordingly, the total substrate requirement was 28.8 m^3 (12 × 2.4 m^3). The mixed substrates had a measured bulk

density of approximately 0.6 $t \cdot m^{-3}$. After the plots were filled, they were irrigated to allow the substrate to settle, and one-year-old blueberry (Vaccinium corymbosum L.) plants (approximately 30 cm tall) were transplanted at uniform spacing. One-year-old nursery plants were chosen because they represent a uniform developmental stage with well-established root systems, which ensures consistent transplanting success and minimizes age-related variability in growth responses, as commonly adopted in blueberry cultivation trials [16, 20]. Throughout the cultivation period, substrate moisture was maintained at approximately 60–70% by regular irrigation with deionised water based on gravimetric monitoring, ensuring consistent moisture conditions for both plant growth and microbial activity. No additional chemical fertilizers or external nutrient supplements were applied during the cultivation period, so that the observed effects could be attributed solely to the SMS amendments. Standard agronomic practices including irrigation, weed control, and pest management were applied uniformly to all plots throughout the growing period. Substrate samples from CKS and CKM were designated accordingly for subsequent physicochemical and microbiological analyses. Blueberry seedlings were transplanted on May 15, 2025.

Text S2. Sample collection and determination of nutritional elements in rhizosphere soil

Rhizosphere soil samples were collected at four time points (15 June, 15 September, 15 December 2025, and 15 March 2026). At each sampling time, three blueberry plants were randomly selected from each plot. After gently removing the bulk soil from the roots by shaking, the soil tightly adhering to the root surface (0 – 2 mm) was carefully brushed off using a sterile brush and collected as rhizosphere soil. The rhizosphere soil samples from the three plants within the same plot were pooled to form one composite sample per replicate plot. All samples were placed in sterile plastic bags, transported to the laboratory on ice, and processed within 24 h. A portion of each fresh sample was stored at 4 ℃ in sealed polyethylene bags for enzyme activity assays, with all enzyme analyses completed within 7 days of collection to minimize activity loss. The remainder of each sample was air-dried at room temperature (25 ± 2 ℃) for 7 - 10 days until constant weight, ground to pass through a 2-mm sieve, and stored in sealed containers at room temperature in a desiccator for subsequent physicochemical analyses. All analyses were performed in triplicate for each sample.

Soil organic carbon (OC) was determined by the Walkley-Black dichromate oxidation method [22]. Total nitrogen (TN) was measured by the modified Kjeldahl digestion and distillation method according to ISO 11261:1995 [23]. Hydrolysis nitrogen (HN) was determined by the alkaline hydrolysis diffusion method [24]: briefly, 2.0 g of air-dried soil was digested with 10 mL of 1.0 M NaOH at 40 ℃ for 24 h, and the released NH_3 was absorbed in 2 % boric acid indicator solution, followed by titration with 0.01 M H_2SO_4. Total phosphorus (TPH) was determined by H_2SO_4-$HClO_4$ digestion followed by molybdenum blue colorimetry [25]. Available phosphorus (APH) was extracted with 0.5 M $NaHCO_3$ (pH 8.5) using the Olsen method [26] and quantified by molybdenum blue colorimetry. Total potassium (TPO) was determined by atomic emission spectrometry (ICPS-7500, Shimadzu, Japan) after HF-$HClO_4$ digestion [27]. Available potassium (APO) was extracted with 1 M ammonium acetate (pH 7.0) and determined by flame photometry [28]. Exchangeable sodium (Na^+), calcium (Ca^{2+}), and magnesium (Mg^{2+}) were extracted with 0.1 M $BaCl_2$ and quantified by flame photometry [29]. Electrical conductivity (EC) was

measured in a soil - water suspension (1:2.5, w/v) using a Hanna Instruments HI9813-6 portable pH/EC meter (Woonsocket, RI, USA) [30]. Soil moisture content was determined gravimetrically by oven-drying at 105 ° C to constant weight according to ISO 11465:1993 [31].

Text S3. Determination of enzyme activities and biochemical components in rhizosphere soil

Cellulase and acid xylanase activities were assayed using the 3,5-dinitrosalicylic acid (DNS) method [32]. For cellulase, 1 g of fresh soil was incubated with 5 mL of 1% carboxymethyl cellulose (CMC) in acetate buffer (pH 5.5) at 37 °C for 24 h; for xylanase, 1% beechwood xylan was used as substrate. The reducing sugars released were measured at 540 nm. Laccase activity was determined by oxidation of 2 mM ABTS in acetate buffer (pH 5.0) at 420 nm after 1 h incubation [33]. Peroxidase activity was measured using pyrogallol as substrate (oxidation to purpurogallin, absorbance at 430 nm) [34]. Protease activity was assayed using 1% casein in Tris–HCl buffer (pH 7.4) at 37 °C for 2 h, and the released amino acids were quantified with Folin–Ciocalteu reagent [35]. Lipase activity was determined using p-nitrophenyl palmitate (pNPP) as substrate; after incubation at 37 °C for 1 h, the released p-nitrophenol was measured at 410 nm [36]. Enzyme activities are expressed in nkat per gram of dry soil (nkat g^{-1}) for cellulase, xylanase, laccase, protease, and lipase. Peroxidase activity is expressed in 10^{-7} $kg{\cdot}kg^{-1}{\cdot}s^{-1}$, as per the cited method [34].

Lignin, hemicellulose, and cellulose contents were determined using the detergent fiber analysis system according to the Van Soest method [37]; this method quantifies total lignin content as acid detergent lignin (ADL), representing the bulk insoluble lignocellulosic fraction in soil. Glucose concentrations were determined by high-performance anion-exchange chromatography with pulsed amperometric detection (HPAEC-PAD) using a CarboPac PA10 column and NaOH gradient elution, following centrifugation and filtration of the soil slurry samples [38].

The lignin phenolic hydroxyl ratio was measured using the CuO oxidation method followed by gas chromatographic analysis of vanillyl, syringyl, and cinnamyl phenols [39]; this method specifically reflects the degree of oxidative modification of lignin structure rather than total lignin content, and was used in addition to the Van Soest method to assess the chemical alteration of lignin during decomposition. The phenolic hydroxyl ratio was calculated relative to a standard lignin preparation [40].

Text S4. Soil Microbial Community Analysis

The primer sequences and PCR conditions for microbial community analysis are provided in the main text (Section 2.4). Total genomic DNA was extracted from 0.5 g of rhizosphere soil using the FastDNA Spin Kit for Soil (MP Biomedicals, Irvine, CA, USA) following the manufacturer's instructions. Following PCR amplification, amplicons were purified, quantified using a Qubit fluorometer (Thermo Fisher Scientific, Waltham, MA, USA), and pooled in equimolar ratios. Sequencing libraries were constructed using the NEBNext Ultra DNA Library Prep Kit (New England Biolabs, Ipswich, MA, USA). Paired-end sequencing (2 × 250 bp) was performed on an Illumina HiSeq 2500 platform (Illumina, San Diego, CA, USA) [43].

Raw demultiplexed sequences were quality-filtered, denoised, and merged into amplicon sequence variants (ASVs) using the DADA2 pipeline within QIIME2 (version 2021.11) [44]. Taxonomic classification was performed using the SILVA 138 database [45] for bacteria and the UNITE 8.0 database [46] for fungi. Alpha diversity indices were calculated using QIIME2. Beta diversity was evaluated by principal coordinates analysis (PCoA) based on weighted UniFrac distances [47].

For each treatment, three independent biological replicates (three replicate plots) were analyzed. No technical replicates were performed.

Table S1. Kendall integrated correlation among Height increasment of blueberry plants compared with rhizosphere microbial community structure, nutrient elements, soil enzyme activities, lignocellulose components, and lignin phenolic hydroxyl ratio, and other physicochemical properties in blueberry rhizosphere soil.

	Chloroflexota	*Acidobacteriota*	*Bacillota*	*Actinobacteriota*	*Myxococcota*	*Planctomycetota*	*Streptomyces*	*Kribbella*	*Ktedonobacter*	*Rhodanobacter*
Height increasment	0.281**	0.343**	0.247*	-0.508**	0.242*	0.239*	-0.455**	-0.243*	-0.224*	-0.288**
	Acidibacter	*Mycobacterium*	*Bauldia*	*Hyphomicrobium*	*Paraburkholderia*	*Chitinophaga*	*Bradyrhizobium*	-	-	
Height increasment	0.245*	-0.332**	0.337**	0.292**	-0.406**	-0.460**	-0.221*	-	-	
	Scedosporium	*Rasamsonia*	*Penicillium*	*Phialemonium*	*Coniochaeta*	*Arthrographis*	*Acremoniopsis*	*Trichoderma*	*Pseudeurotium*	
Height increasment	0.226*	0.383**	-0.295**	-0.248*	-0.313**	0.272**	-0.318**	-0.337**	-0.321**	
	Cellulase	Soil acid xylanase	Laccase	Peroxidase	Protease	Slipase	Lignin	Cellilose	Soil lignin phenolic	-
Height increasment	0.447**	0.501**	0.524**	0.452**	0.454**	0.788**	-0.324**	0.447**	0.533**	-
	OC	TN	HN	TPH	APH	TPO	APO	Na^{+}	Ca^{2+}	Mg^{2+}
Height increasment	0.533**	0.397**	0.344**	0.445**	0.291**	0.419**	0.263*	0.467**	-0.585**	-0.578**
	Soil EC	Soil moisture content	-	-	-	-	-	-	-	-
Height increasment	-0.289**	0.629**	-	-	-	-	-	-	-	-

Notes: OC: Soil organic carbon, TN: Total nitrogen, HN:Alkaline hydrolysis nitrogen , TPH: Total phosphorus, APH: Available phosphorus,

TPO: Total potassium, APO: Available potassium, Na^+: Sodium ion, Ca^{2+}: Calcium ion, Mg^{2+}: Magnesium ion, Soil EC: Soil electrical conductivity.

Table S2. Spearman integrated correlation among Height increasment of blueberry plants compared with rhizosphere microbial community structure, nutrient elements, soil enzyme activities, lignocellulose components, and lignin phenolic hydroxyl ratio, and other physicochemical properties in blueberry rhizosphere soil.

	Actinobacteriota	*Bacillota*	*Acidobacteriota*	*Chloroflexota*	*Myxococcota*	*Planctomycetota*	*Streptomyces*	*Alicyclobacillus*	*Ktedonobacter*	*Kribbella*
Height increasment	-0.693**	0.340*	0.479**	0.398**	0.373**	0.341*	-0.644**	0.293*	-0.285*	-0.323*
	Rhodanobacter	*Bauldia*	*Mycobacterium*	*Acidibacter*	*Hyphomicrobium*	*Paraburkholderia*	*Chitinophaga*	*Bradyrhizobium*		
Height increasment	-0.367*	0.495**	-0.478**	0.373**	0.405**	-0.564**	-0.658**	-0.346*		
	Scedosporium	*Rasamsonia*	*Phialemonium*	*Penicillium*	*Acremoniopsis*	*Arthrographis*	*Coniochaeta*	*Trichoderma*	*Pseudeurotium*	
Height increasment	0.344*	0.540**	-0.333*	-0.394**	-0.457**	0.399**	-0.442**	-0.473**	-0.444**	
	Cellulase	Soil acid xylanase	Laccase	Peroxidase	Protease	Slipase	Cellilose	Soil lignin phenolic	-	
Height increasment	0.622**	0.670**	0.716**	0.621**	0.627**	0.931**	-0.470**	0.715**	-	-
	OC	TN	HN	TPH	APH	TPO	APO	Na^{+}	Ca^{2+}	Mg^{2+}
Height increasment	0.563**	0.517**	0.629**	0.412**	0.589**	0.365*	0.626**	-0.765**	-0.775**	-0.422**
	Soil EC	Soil moisture content								
Height increasment	-0.419**	0.803**								

Notes: OC: Soil organic carbon, TN: Total nitrogen, HN:Alkaline hydrolysis nitrogen , TPH: Total phosphorus, APH: Available phosphorus, TPO: Total potassium, APO: Available potassium, Na^{+}: Sodium ion, Ca^{2+}: Calcium ion, Mg^{2+}: Magnesium ion, Soil EC: Soil electrical conductivity.

Table S3. Eigenvectors (factor loadings) of the measured soil properties, enzyme activities, lignocellulose components, and microbial taxa on the first eight principal components (PC1 – PC8) derived from principal component analysis (PCA).

Variables	Principal Components							
	PC1	PC2	PC3	PC4	PC5	PC6	PC7	PC8
Actinobacteriota	-0.709	0.092	0.630	0.092	0.116	-0.013	-0.064	-0.108
Bacillota	0.518	0.295	0.202	-0.480	-0.411	0.150	0.217	-0.048
Acidobacteriota	0.170	-0.783	-0.365	0.272	0.124	-0.130	0.001	0.003
Chloroflexota	0.177	-0.823	-0.210	0.280	0.043	-0.030	0.087	-0.074
Myxococcota	0.060	-0.639	-0.544	0.272	0.011	0.092	-0.184	-0.004
Planctomycetota	0.466	0.172	0.163	-0.379	-0.314	-0.096	0.262	0.440
Streptomyces	-0.660	0.010	0.658	0.086	0.015	0.022	-0.071	-0.092
Kribbella	-0.660	-0.226	0.496	0.213	0.025	0.202	0.032	-0.011
Ktedonobacter	-0.511	-0.238	0.617	0.053	0.121	0.167	0.029	-0.130
Rhodanobacter	-0.253	0.557	0.183	0.031	0.217	-0.529	-0.187	-0.129
Mycobacterium	-0.542	0.636	0.219	0.158	0.123	-0.325	0.071	0.042
Bauldia	0.244	-0.722	-0.502	0.131	0.197	-0.086	-0.089	-0.013
Hyphomicrobium	0.639	0.518	-0.129	-0.101	0.399	0.123	-0.074	-0.006
Acidibacter	0.428	-0.091	-0.344	-0.302	0.218	0.400	0.177	-0.292
Rhizobium	-0.370	0.344	-0.552	0.236	0.060	0.015	0.384	0.376
Paraburkholderia	-0.648	0.417	0.304	0.001	0.297	0.060	0.127	0.085
Chitinophaga	-0.554	0.354	0.110	0.391	-0.136	0.319	0.010	0.173
Bradyrhizobium	-0.410	0.195	-0.079	0.109	0.614	0.492	0.032	0.288
Basidiomycota	-0.461	-0.190	-0.148	0.663	-0.281	0.037	-0.040	0.009
Sordariales_gen_Incertae_sedis	0.492	0.002	0.193	0.342	0.279	0.087	-0.187	0.058
Rasamsonia	0.447	0.155	0.303	-0.134	-0.338	-0.196	-0.403	0.346
Penicillium	-0.409	0.235	-0.215	0.031	0.336	-0.584	0.181	-0.189
Phialemonium	-0.255	0.383	-0.247	0.524	-0.263	0.152	0.077	-0.024
Arthrographis	0.580	0.173	0.060	-0.229	0.006	0.295	0.395	-0.334
Acremoniopsis	-0.290	0.408	-0.198	0.055	0.274	-0.461	0.260	0.106
Trichoderma	-0.576	-0.304	0.136	-0.254	0.300	0.228	-0.014	0.318
Pseudeurotium	-0.572	-0.372	0.604	0.014	0.053	0.056	0.002	-0.178
Clitopilus	-0.260	0.298	-0.035	0.754	-0.246	0.251	0.029	-0.061
Cellulase	0.885	0.107	0.314	0.264	0.062	-0.055	0.079	0.045
Soilacidxylanase	0.900	0.120	0.171	0.331	-0.049	0.039	0.038	-0.018
Laccase	0.869	0.189	-0.111	0.163	-0.127	-0.135	0.022	-0.127
Peroxidase	0.851	0.372	0.109	0.235	0.177	0.035	-0.016	-0.002
Protease	0.869	0.019	0.250	0.312	0.086	-0.026	0.106	0.072
Slipase	0.821	-0.244	-0.044	0.215	0.261	-0.085	-0.080	0.047
Lignin	-0.118	0.782	-0.421	-0.238	0.041	0.205	-0.241	-0.083
Cellilose	-0.320	0.582	-0.529	-0.320	-0.140	0.117	-0.276	-0.079

OC	0.744	0.120	0.084	0.111	0.055	0.078	-0.177	-0.003
TN	0.805	0.436	0.094	0.219	0.033	0.085	-0.013	-0.063
HN	0.891	0.202	0.184	0.265	-0.073	-0.022	0.073	0.026
APH	0.864	0.217	0.344	0.129	-0.035	-0.068	0.072	0.010
APO	0.776	0.550	0.062	0.112	0.166	0.056	-0.063	-0.006
Na^+	-0.806	0.143	-0.222	0.228	-0.347	-0.082	0.107	-0.056
Ca^{2+}	-0.557	0.739	-0.187	-0.051	0.073	0.163	-0.122	-0.013
Mg^{2+}	0.127	0.948	-0.106	0.028	-0.116	0.036	-0.052	-0.079
Soil EC	-0.213	0.887	-0.187	0.235	0.089	0.015	-0.025	-0.076
Soil moisture content	0.878	-0.212	0.118	-0.211	0.114	0.080	-0.051	0.113

Notes: OC: Soil organic carbon, TN: Total nitrogen, HN:Alkaline hydrolysis nitrogen , APH: Available phosphorus, APO: Available potassium, Na^+: Sodium ion, Ca^{2+}: Calcium ion, Mg^{2+}: Magnesium ion, Soil EC: Soil electrical conductivity.

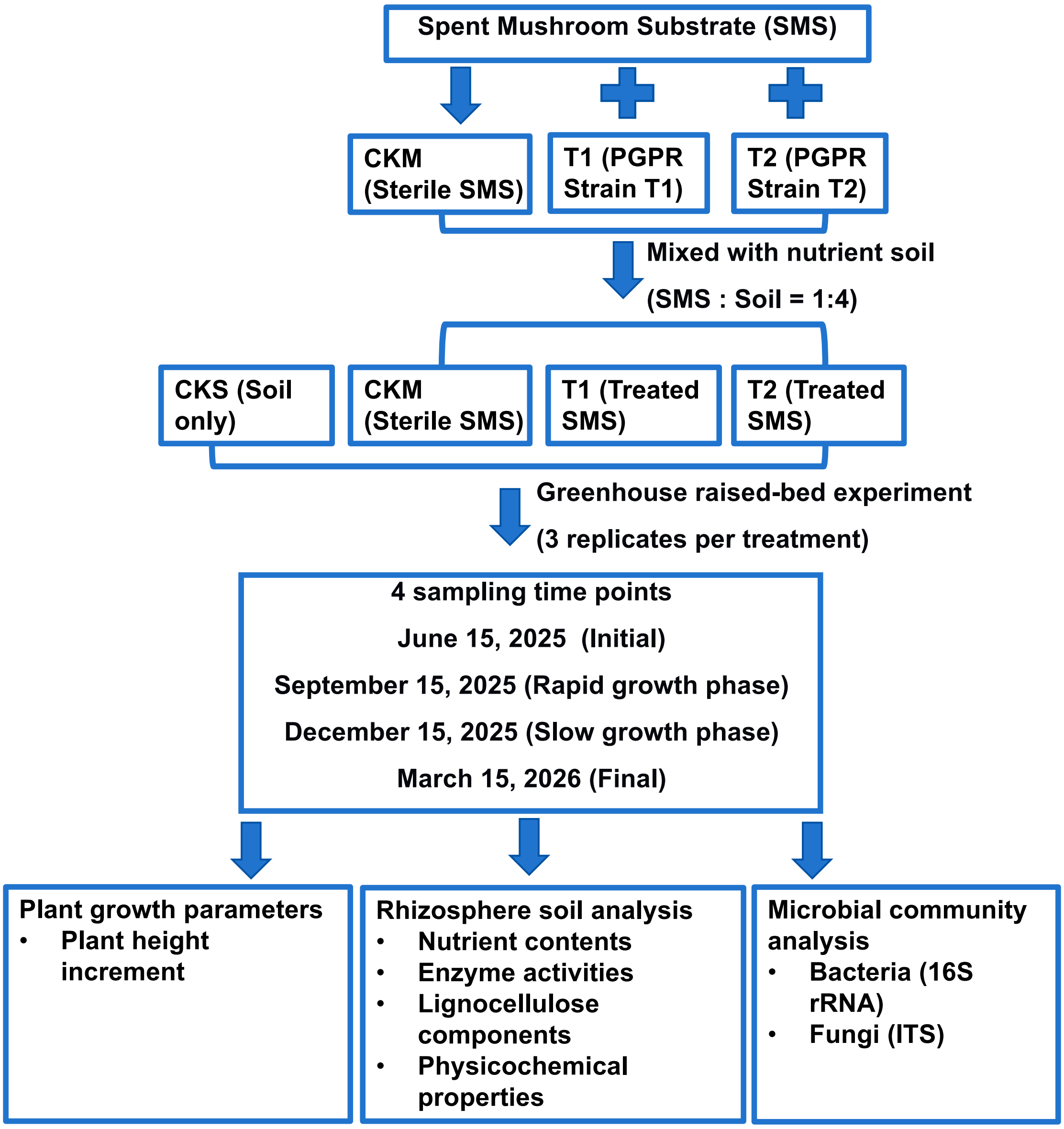

Figure S1. Schematic diagram of the experimental design. CKS: conventional cultivation soil (control); CKM: conventional soil and sterilized mushroom residue (1:4, v/v); T1: sterilized mushroom residue treated with PGPR strain T1 mixed with conventional soil (1:4, v/v); T2: sterilized mushroom residue treated with PGPR strain T2 mixed with conventional soil (1:4, v/v). SMS: spent mushroom substrate; PGPR: plant growth-promoting rhizobacteria.

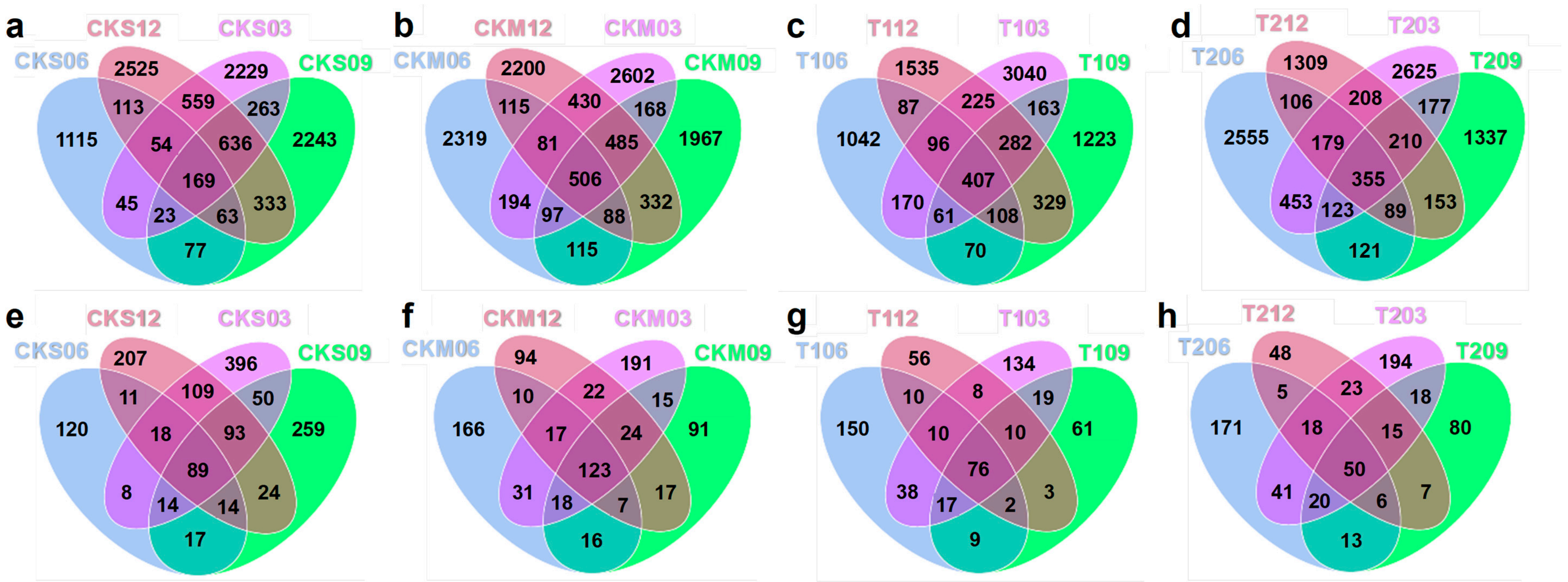

Figure S2 Venn diagram showing the distribution of unique and shared OTUs of the microbial community in the blueberry rhizosphere soil at four sampling times under different treatments (CKS, CKM, T1, and T2) based on high-throughput sequencing.

Notes: CKS06, CKS09, CKS12, and CKS03 refer to the CKS rhizosphere soil samples at different sampling periods (June in 2025, September in 2025, December in 2025, and March in 2026), respectively. CKM06, CKM09, CKM12, and CKM03 refer to the CKM rhizosphere soil samples at different sampling periods (June in 2025, September in 2025, December in 2025, and March in 2026), respectively. T106, T109, T112, and T103 refer to the T1 rhizosphere soil samples at different sampling periods (June in 2025, September in 2025, December in 2025, and March in 2026), respectively. T206, T209, T212, and T203 refer to the T2 rhizosphere soil samples at different sampling periods (June in 2025, September in 2025, December in 2025, and March in 2026), respectively. a-d represent the veen diagram of bacteria, while e-h represent the veen diagram of fungi.

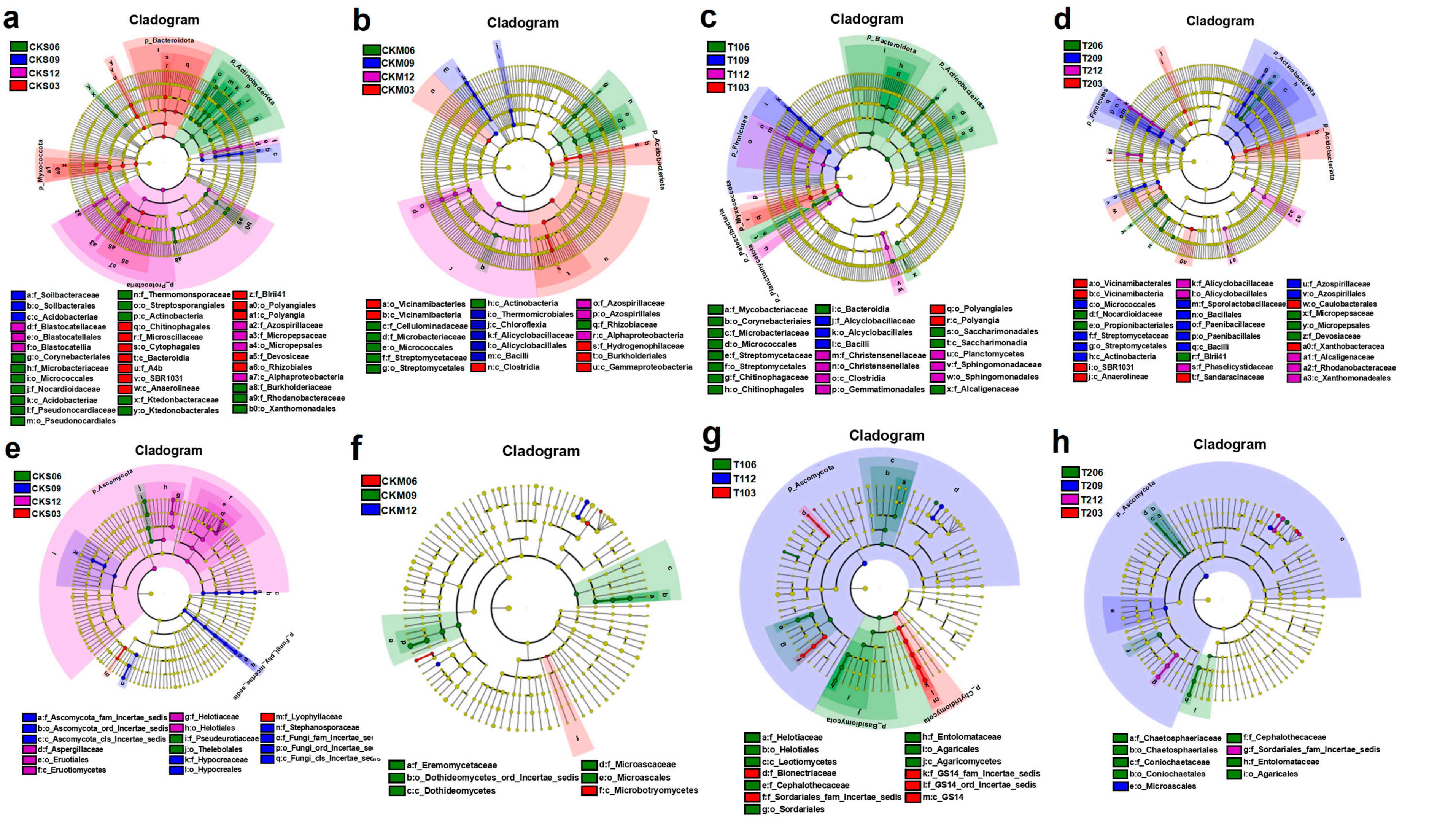
a
Cladogram
CKS06
CKS09
CKS12
CKS03
a:f_Soilbacteraceae
b:o_Soilbacteraies
c:c_Acidobacteriae
d:f_Blastocatellaceae
e:o_Blastocatellales
f:o_Blastocatellia
g:o_Corynebacteriales
h:f_Microbacteriaceae
i:o_Micrococcales
j:f_Nocardioidaceae
k:c_Acidobacteriae
l:f_Pseudonocardiaceae
m:o_Pseudonocardiales
n:f_Thermomonsporaceae
o:o_Streptosporangiales
p:c_Actinobacteria
q:o_Chitinophagales
r:f_Microscillaceae
s:o_Cytophagales
t:c_Bacteroidia
u:f_A4b
v:o_SBR1031
w:c_Anaerolineae
x:f_Ktedonbacteraceae
y:o_Ktedonobacterales
z:f_Blrii41
a0:o_Polyangiales
a1:c_Polyangia
a2:f_Azospirillaceae
a3:f_Micropepsaceae
a4:o_Micropepsales
a5:f_Devosiceae
a6:o_Rhizobiales
a7:c_Alphaproteobacteria
a8:f_Burkholderiaceae
a9:f_Rhodanobacteraceae
b0:o_Xanthomonadales
b
Cladogram
CKM06
CKM09
CKM12
CKM03
a:o_Vicinamibacterles
b:c_Vicinamibacteria
c:f_Celluluminadaceae
d:f_Microbacteriaceae
e:o_Micrococcales
f:f_Streptomycetaceae
g:o_Streptomycetales
h:c_Actinobacteria
i:o_Thermomicrobiales
j:c_Chloroflexia
k:f_Alicyclobacillaceae
l:o_Alicyclobacillales
m:c_Bacilli
n:c_Clostridia
o:f_Azospirillaceae
p:o_Azospirillales
q:f_Rhizobiaceae
r:c_Alphaproteobacteria
s:f_Hydrogenophilaceae
t:o_Burkholderiales
u:c_Gammaproteobacteria
c
Cladogram
T106
T109
T112
T103
a:f_Mycobacteriaceae
b:o_Corynebacteriales
c:f_Microbacteriaceae
d:o_Micrococcales
e:f_Streptomycetaceae
f:o_Streptomycetales
g:f_Chitinophagaceae
h:o_Chitinophagales
i:c_Bacteroidia
j:f_Alcyclobacillaceae
k:o_Alcyclobacillales
l:c_Bacilli
m:f_Christensenellaceae
n:o_Christensenellales
o:c_Clostridia
p:o_Gemmatimonadales
q:o_Polyangiales
r:c_Polyangia
s:o_Saccharimonadales
t:c_Saccharimonadia
u:c_Planctomycetes
v:f_Sphingomonadaceae
w:o_Sphingomonadales
x:f_Alcaligenaceae
d
Cladogram
T206
T209
T212
T203
a:o_Vicinamibacterales
b:c_Vicinamibacteria
c:o_Micrococcales
d:f_Nocardioidaceae
e:o_Propionibacteriales
f:f_Streptomycetaceae
g:o_Streptomycetales
h:c_Actinobacteria
i:o_SBR1031
j:c_Anaerolineae
k:f_Alicyclobacillaceae
l:o_Alicyclobacillales
m:f_Sporolactobacillaceae
n:o_Bacillales
o:f_Paenibacillaceae
p:o_Paenibacillales
q:c_Bacilli
r:f_Blrii41
s:f_Phaselicystidaceae
t:f_Sandaracinaceae
u:f_Azospirillaceae
v:o_Azospirillales
w:o_Caulobacterales
x:f_Micropepsaceae
y:o_Micropepsales
z:f_Devosiaceae
a0:f_Xanthobacteracea
a1:f_Alcaligenaceae
a2:f_Rhodanobacteraceae
a3:c_Xanthomonadeales
e
Cladogram
CKS06
CKS09
CKS12
CKS03
a:f_Ascomycota_fam_Incertae_sedis
b:o_Ascomycota_ord_Incertae_sedis
c:c_Ascomycota_cls_Incertae_sedis
d:f_Aspergillaceae
e:o_Erutiales
f:c_Eruotiomycetes
g:f_Helotiaceae
h:o_Helotiales
i:f_Pseudeurotiaceae
j:o_Thelebolales
k:f_Hypocreaceae
l:o_Hypocreales
m:f_Lyophyllaceae
n:f_Stephanosporaceae
f
Cladogram
CKM06
CKM09
CKM12
a:f_Eremomycetaceae
b:o_Dothideomycetes_ord_Incertae_sedis
c:c_Dothideomycetes
d:f_Microascaceae
e:o_Microascales
f:c_Microbotryomycetes
g
Cladogram
T106
T112
T103
a:f_Helotiaceae
b:o_Helotiales
c:c_Leotiomycetes
d:f_Bionectriaceae
e:f_Cephalothecaceae
f:f_Sordariales_fam_Incertae_sedis
g:o_Sordariales
h:f_Entolomataceae
i:o_Agaricales
j:c_Agaricomycetes
k:f_GS14_fam_Incertae_sedis
l:f_GS14_ord_Incertae_sedis
m:c_GS14
h
Cladogram
T206
T209
T212
T203
a:f_Chaetosphaeriaceae
b:o_Chaetosphaeriales
c:f_Coniochaetaceae
b:o_Coniochaetales
e:o_Microascales
f:f_Cephalothecaceae
g:f_Sordariales_fam_Incertae_sedis
h:f_Entolomataceae
i:o_Agaricales

Figure S3. LEfSe evolutionary branch diagram of the microbial community in the blueberry rhizosphere soil at four sampling times under different treatments (CKS, CKM, T1, and T2) based on high-throughput sequencing.

Notes: CKS06, CKS09, CKS12, and CKS03 refer to the CKS rhizosphere soil samples at different sampling periods (June in 2025, September in 2025, December in 2025, and March in 2026), respectively. CKM06, CKM09, CKM12, and CKM03 refer to the CKM rhizosphere soil samples at different sampling periods (June in 2025, September in 2025, December in 2025, and March in 2026), respectively. T106, T109, T112, and T103 refer to the T1 rhizosphere soil samples at different sampling periods (June in 2025, September in 2025, December in 2025, and March in 2026), respectively. T206, T209, T212, and T203 refer to the T2 rhizosphere soil samples at different sampling periods (June in 2025, September in 2025, December in 2025, and March in 2026), respectively. a-d represent the veen diagram of bacteria, while e-h represent the veen diagram of fungi.

a

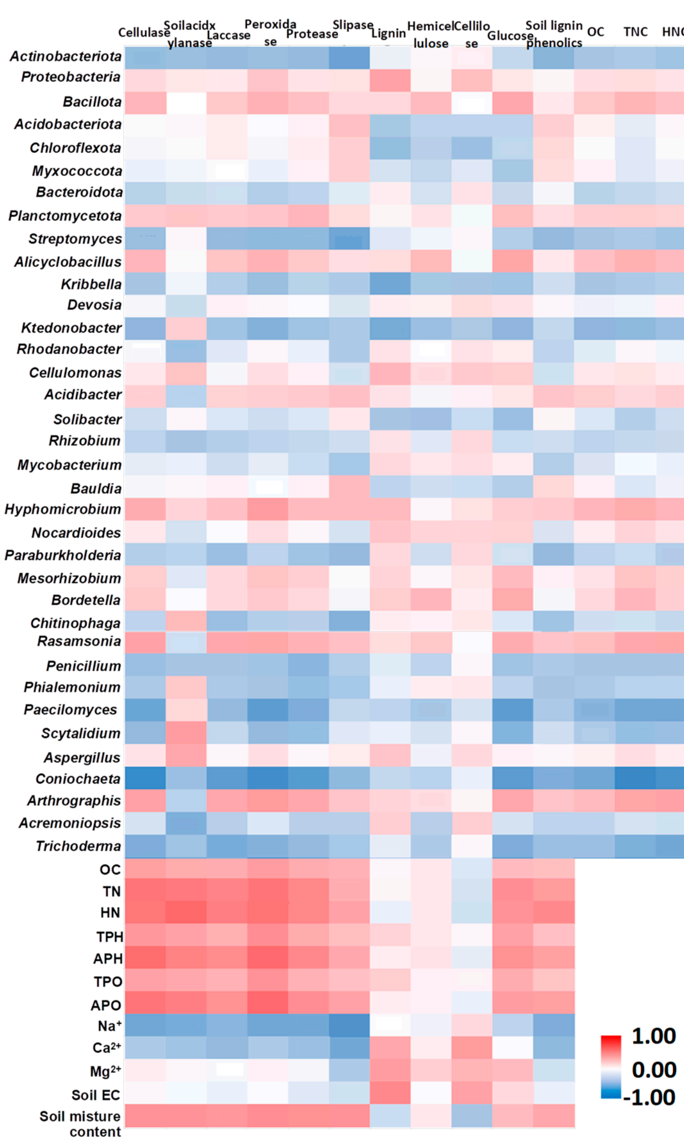
Cellulase
Soilacidxylanase
Laccase
Peroxidase
Protease
Slipase
Lignin
Hemicellulose
Cellulose
Glucose
Soil lignin phenolics
OC
TNC
HNC
TPHC
APHC
TPOC
APOC
Na+
Ca2+
Mg2+
Soil EC
Soil moisture content
Actinobacteriota
Proteobacteria
Bacillota
Acidobacteriota
Chloroflexota
Myxococcota
Bacteroidota
Planctomycetota
Streptomyces
Alicyclobacillus
Kribbella
Devosia
Ktedonobacter
Rhodanobacter
Cellulomonas
Acidibacter
Solibacter
Rhizobium
Mycobacterium
Bauldia
Hyphomicrobium
Nocardioides
Paraburkholderia
Mesorhizobium
Bordetella
Chitinophaga
Rasamsonia
Penicillium
Phialemonium
Paecilomyces
Scytalidium
Aspergillus
Coniochaeta
Arthrographis
Acremoniopsis
Trichoderma
OC
TN
HN
TPH
APH
TPO
APO
Na+
Ca2+
Mg2+
Soil EC
Soil misture content
1.00
0.00
-1.00

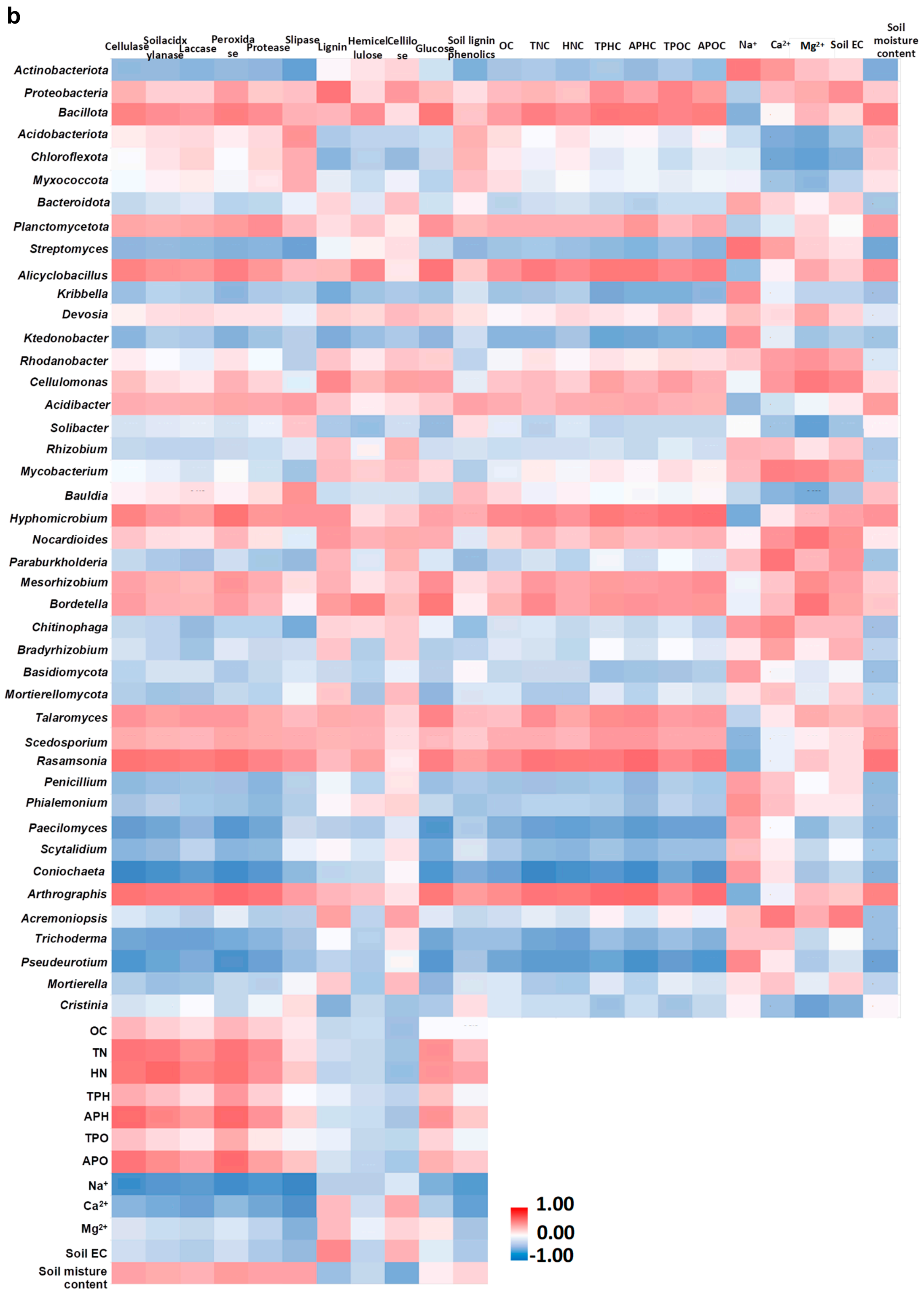

Figure S4 Integrated correlation heatmap showing associations among rhizosphere microbial community structure, nutrient elements, soil enzyme activities, lignocellulose components, and lignin phenolic hydroxyl ratio, and other physicochemical properties in blueberry rhizosphere soil.

Kendall Correlation (a) and Spearman Correlation (b) were shown by integrated correlation heatmap.The color scale represents the correlation coefficient, ranging from-1 (negative correlation, blue) to +1 (positive correlation, red). Data are presented from three biological replicates (n = 3).

Note: OC: Soil organic carbon, TN: Total nitrogen, HN:Alkaline hydrolysis nitrogen , TPH: Total phosphorus, APH: Available phosphorus, TPO: Total potassium, APO: Available potassium, Na^{+}: Sodium ion, Ca^{2+}: Calcium ion, Mg^{2+}: Magnesium ion, Soil EC: Soil electrical conductivity.
